# Supplementary material for: A Cohort Study Comparing Women with Autism Spectrum Disorder with and without Generalized Joint Hypermobility
Source: Behav Sci (Basel). 2018 Mar 17;8(3):35. doi: 10.3390/bs8030035 (PMC5867488; doi:10.3390/bs8030035)
Supplement: Supplementary file 1 [file behavsci-08-00035-s001.zip › Supplementary_Files/S1.pdf]

# SURVEY: The Relationship of Epilepsy, Endocrine Disorders, and Immune Dysregulation in Adult Women with Autism

---

## Consent Waiver

**RESEARCH STUDY INFORMATION**

**Study to be Conducted at:**
**Greenville Health System**
**Patewood Medical Campus**
**200 Patewood Dr.**
**Suite 200A**
**Greenville, SC 29615**

**Principal Investigator:**
**Manuel Casanova, MD**

**Co-investigator:**
**Emily Casanova, PhD**

**Introduction:**
**You are being asked to participate in a research study. The Institutional Review Board of the Greenville Health System has reviewed this study for the protection of the rights of human participants in research studies, in accordance with federal and state regulations. However, before you choose to be a research participant, it is important that you read the following information and ask as many questions as necessary to be sure that you understand what your participation will involve.**

**Purpose and Procedures:**
**The purpose of this research study is to investigate a relationship between epilepsy and hormone/immune disorders in women with Autism Spectrum Disorder (ASD). You are being asked to participate because you are either a woman with an ASD over the age of 25 or you are the legal guardian of a**

**woman who fits these criteria. If you or your ward don't have a professional ASD diagnosis but suspect the presence of an ASD, we encourage you to participate too. Please also consider participating regardless of whether you or your ward have any of the above listed medical conditions (epilepsy, hormonal, immune), as we are hoping to also better understand the frequency of these conditions in the female population of the spectrum.**

**We plan to enroll at least several thousand people in this study. Your participation will involve answering an online survey to the best of your abilities. This should take approximately 15-25 minutes.**

## Possible Risks and Benefits:

**There are no known medical risks related to participation in this study. The greatest risk is the possible release of your personal information with the investigators. However, this survey is anonymous and we will not be collecting any identifying information. Your survey answers are considered confidential, but absolute confidentiality cannot be guaranteed. This study may result in presentations and publications. There are no direct benefits to you that would result from your participation in the study.**

**Although you will not receive compensation for participating in this study, this research may help us to understand these conditions better. This knowledge can help scientists and doctors develop better treatments for co-occurring medical conditions in ASD that may affect you or your family.**

## Voluntary Participation:

**Participation in this study is completely voluntary (your choice). You may refuse to participate or withdraw prior to submitting your survey. If you refuse to participate or stop the survey, you will not be penalized or lose any benefits. However, because your submission is anonymous, we will not be able to remove your data from the study afterwards. So please be certain you want to participate prior to submission. Your decision will not affect your relationship with the investigators or any relationship you may have with the Greenville Health System.**

## Contact for Questions:

**For more information concerning this study and research-related risks or injuries, or to give comments or express concerns or complaints, you may contact the principal investigator, *Manuel Casanova*, at (864) 454-4595.**

**You may also contact a representative of the Institutional Review Board of the Greenville Health System for information regarding your rights as a participant involved in a research study or to give comments or express concerns, complaints, or offer input. You may obtain the name and number of this person by calling (864) 455-8997.**

**An additional survey about your experience with this informed consent process is located at the following website: https://www.surveymonkey.com/s/T5C86P8 Participation in the above survey is completely anonymous and voluntary and will not affect your relationship with the Greenville Health System. If you would like to have a paper copy of this survey, please tell the principle investigator.**

**Page exit logic:** Skip / Disqualify Logic**IF:** Question "Do you agree to participate in this survey study?" #1 is one of the following answers ("Yes, I am a woman on the autism spectrum who is 25 years or older and I consent to participate in this study.","Yes, I am a legal guardian of a woman on the spectrum who is 25 years or older and I consent to participate in this study.") **THEN:** Jump to page 2 - General Information Flag response as complete

**Page exit logic:** Skip / Disqualify Logic**IF:** Question "Do you agree to participate in this survey study?" #1 is one of the following answers ("No, I do not consent.") **THEN:** Jump to page 9 - Flag response as complete

**Logic: Show/hide trigger exists.**

**1) Do you agree to participate in this survey study?***

( ) Yes, I am a woman on the autism spectrum who is 25 years or older and I consent to participate in this study.

( ) Yes, I am a legal guardian of a woman on the spectrum who is 25 years or older and I consent to participate in this study.

( ) No, I do not consent.

---

# General Information

## Here we want to get some background information and details about diagnoses.

**2) Relationship to adult on the spectrum:**

( ) Self

( ) Mother

( ) Father

( ) Sibling

( ) Other, please specify:: ____________________________________________

### 3) Current age (in years) of the adult on the spectrum. (Please do <u>NOT</u> provide birth dates.)

____________________________________________

**Logic: Show/hide trigger exists.**

**4) Does the adult on the spectrum have a professional diagnosis of an autism spectrum disorder or a related condition?**

( ) Yes

( ) Informally diagnosed (e.g., self-diagnosis)

( ) No

( ) Other, please specify:: ____________________________________________

**Logic: Hidden unless: Question "Does the adult on the spectrum have a professional diagnosis of an autism spectrum disorder or a related condition?" #4 is one of the following answers ("Yes")**

## 5) If professionally diagnosed, what is the formal diagnosis?

____________________________________________

**Logic: Hidden unless: Question "Does the adult on the spectrum have a professional diagnosis of an autism spectrum disorder or a related condition?" #4 is one of the following answers ("Informally diagnosed (e.g., self-diagnosis)")**

## 6) If informally diagnosed (e.g., self-diagnosis), what is the suspected diagnosis?

____________________________________________

**Logic: Show/hide trigger exists.**

**7) Does the adult on the spectrum have a professional diagnosis of another neurodevelopmental, psychiatric, or neurological condition (e.g., ADHD, bipolar disorder, etc)?**

( ) Yes

( ) No

( ) Don't know

( ) Other, please specify:: ____________________________________________

**Logic: Hidden unless: Question "Does the adult on the spectrum have a professional diagnosis of another neurodevelopmental, psychiatric, or neurological condition (e.g., ADHD, bipolar disorder, etc)?" #7 is one of the following answers ("Yes")**

## 8) Please list the diagnoses:

________________________________________

________________________________________

________________________________________

________________________________________

**Logic: Show/hide trigger exists.**

**9) Does the adult on the spectrum have a diagnosis of a *genetic* condition or syndrome (e.g., Fragile X Syndrome, Fetal Alcohol Syndrome, etc.)? (This does not necessarily have to be related to her autism.)**

( ) Yes

( ) Suspected, but unconfirmed

( ) No

( ) Don't know

( ) Other, please specify:: _______________________________________________

**Logic: Hidden unless: Question "Does the adult on the spectrum have a diagnosis of a *genetic* condition or syndrome (e.g., Fragile X Syndrome, Fetal Alcohol Syndrome, etc.)? (This does not necessarily have to be related to her autism.)" #9 is one of the following answers ("Yes","Suspected, but unconfirmed")**

## 10) What is the diagnosis and/or genetic mutation(s) (if known)?

_______________________________________________

**11) Do you have any children diagnosed with EDS?***

( ) Yes, I have at least one child who is also diagnosed with EDS.

( ) No, I have at least one child and s/he doesn't have a diagnosis of EDS

( ) I don't have any children

( ) Don't know

( ) Other, please explain:: _______________________________________________*

**12) Have either of your parents had a diagnosis of EDS or has/had similar symptoms?***

( ) Yes, at least one of my parents has/had a diagnosis of EDS

( ) Yes, at least one of my parents has/had symptoms similar to EDS

( ) No, neither of my parents has/had a diagnosis or symptoms of EDS

( ) Don't know

( ) Other, please explain:: _______________________________________________*

---

# History of Infections & Immune-related Conditions

**13) Any history of hospitalization due to infection during childhood, the teenage years, or adulthood? Check all boxes that apply and leave blank those that do not.**

| | **Childhood (0-9 yrs)** | **Teen (10-17 yrs)** | **Adult (18 yrs & older)** |
|---|---|---|---|
| Yes | [ ] | [ ] | [ ] |
| No | [ ] | [ ] | [ ] |
| Don't know | [ ] | [ ] | [ ] |

**14) Any history of the following conditions during childhood, the teenage years, or adulthood? As above, check all boxes that apply.**

| | **Childhood (0-9 yrs)** | **Teen (10-17 yrs)** | **Adult (18 yrs & older)** |
|---|---|---|---|
| Asthma | [ ] | [ ] | [ ] |
| Frequent ear infections | [ ] | [ ] | [ ] |
| Frequent rhinitis | [ ] | [ ] | [ ] |

| | | | |
|---|---|---|---|
| (inflammation of membranes of the nose) | | | |
| Frequent sinusitis (inflammation of the sinuses) | [ ] | [ ] | [ ] |
| Irritable Bowel Syndrome (IBS) or other gastrointestinal problems | [ ] | [ ] | [ ] |
| Mast Cell Activation Syndrome/Disorder (MCAS/MCAD) | [ ] | [ ] | [ ] |
| Chronic allergies | [ ] | [ ] | [ ] |
| Severe or unusual physical reactions to medications (anaphylaxis, severe drowsiness, rash, etc.) | [ ] | [ ] | [ ] |
| Severe or unusual physical reactions to environmental chemicals (runny nose, coughing, asthma, rash, etc.) | [ ] | [ ] | [ ] |

**15) Does the adult on the spectrum have or is suspected of having an autoimmune condition, such as Diabetes I or Rheumatoid Arthritis? (Here is a list of common autoimmune disorders: http://www.niams.nih.gov/health_info/autoimmune/#2 )**

( ) Yes, professionally diagnosed

( ) Suspected but not professionally diagnosed

( ) No

( ) Don't know

( ) Other, please explain:: _____________________________________________

**16) If the adult on the spectrum has experienced any acute events, such as unusual illnesses, severe reactions to inoculation, etc., or chronic immune-related conditions that you want to tell us about that have not been covered by the three previous questions, please describe below.**

____________________________________________
____________________________________________
____________________________________________
____________________________________________

**17) Is there any other information about the participant's immune function that you would like to tell us that has not already been covered? If so, please write below.**

____________________________________________
____________________________________________
____________________________________________
____________________________________________

---

# Connective Tissue Problems

**This section of the survey addresses issues related to connective tissues, such as joints and skin.**

**Logic: Show/hide trigger exists.**

**18) Does the adult on the spectrum have a diagnosis of Ehlers-Danlos Syndrome (EDS) or Joint Hypermobility Syndrome?**

( ) Yes

( ) No, but suspected

( ) No

( ) Don't know

( ) Other, please explain:: _______________________________________________

**Logic: Hidden unless: Question "Does the adult on the spectrum have a diagnosis of Ehlers-Danlos Syndrome (EDS) or Joint Hypermobility Syndrome?" #18 is one of the following answers ("Yes","No, but suspected")**

**19) If the adult on the spectrum has a form of Ehlers-Danlos (EDS), what type does she have?**

( ) I haven't been diagnosed with EDS - I have a diagnosis of Joint Hypermobility Syndrome

( ) I have EDS Hypermobility Type

( ) I have EDS Classical Type

( ) I have EDS Vascular Type

( ) I have EDS Kyphoscoliosis Type

( ) I have EDS Arthrocalasia Type

( ) I have EDS Dermatosparaxis Type

( ) Other EDS, please specify:: _______________________________________________

**20) Is the adult on the spectrum in any way "double-jointed"?**

( ) Yes, across 2 or more types of joints (e.g., wrists, knees, elbows, etc.)

( ) Yes, in a single type of joint (e.g., knees)

( ) No

( ) Don't know

( ) Other, please explain:: _______________________________________________

**21) Does the adult on the spectrum have any of the following physical issues? Please check all that apply.**

| | Yes | No | Don't know |
|---|---|---|---|
| Bruises easily | [ ] | [ ] | [ ] |
| Has bleeds that are difficult to control | [ ] | [ ] | [ ] |
| Thin skin | [ ] | [ ] | [ ] |
| Scars easily | [ ] | [ ] | [ ] |

**22) Does the adult on the spectrum have any kind of chronic joint paint (arthralgia) or arthritis that is unusual (or at one time was) for her age and is not otherwise due to injury?**

( ) Yes

( ) No

( ) Don't know

( ) Other, please explain:: ____________________________________________

**23) Does the adult on the spectrum have any kind of chronic pain (not otherwise due to injury) not addressed in the previous question?**

( ) Yes

( ) No

( ) Don't know

( ) Other, please explain:: ____________________________________________

**Logic: Hidden unless: (Question "Does the adult on the spectrum have any kind of chronic joint paint (arthralgia) or arthritis that is unusual (or at one time was) for her age and is not otherwise due to injury?" #22 is one of the following answers ("Yes") OR Question "Does the adult on the spectrum have any kind of chronic pain (not otherwise due to injury) not addressed in the previous question?" #23 is one of the following answers ("Yes"))**

**24) Describe the chronic pain she experiences.**

________________________________________________________________________

________________________________________________________________________

________________________________________________________________________

________________________________________________________________________

**25) Is there anything else about connective tissue disorders or chronic pain that the adult may experience that you would like to tell us about? Please write below.**

________________________________________________________________________

________________________________________________________________________

________________________________________________________________________

________________________________________________________________________

---

# Seizure History

**This section of the survey gathers information on seizure and medication history.**

**Logic: Show/hide trigger exists.**

**26) Does the adult on the spectrum have a history of epilepsy that has been diagnosed by a healthcare professional? ("Epilepsy" can be preceded by but does not include fever-related [febrile] seizures.)**

( ) Yes

( ) No

( ) Don't know

( ) Other, please explain:: _________________________________________________

**Logic: Hidden unless: Question "Does the adult on the spectrum have a history of epilepsy that has been diagnosed by a healthcare professional? ("Epilepsy" can be preceded by but does not include fever-related [febrile] seizures.)" #26 is one of the following answers ("Yes")**

## 27) Please describe the types of seizures the individual has experienced or still experiences. Also include any descriptions of fever-related seizures she may have had.

____________________________________________

____________________________________________

____________________________________________

____________________________________________

**Logic: Hidden unless: Question "Does the adult on the spectrum have a history of epilepsy that has been diagnosed by a healthcare professional? ("Epilepsy" can be preceded by but does not include fever-related [febrile] seizures.)" #26 is one of the following answers ("Yes")**

## 28) At approximately what age did the epileptic seizures begin? (If the individual's epilepsy was preceded by fever-related seizures, please give approximate age that non-fever-related seizures began.)

_________________________________________________

**Logic: Show/hide trigger exists. Hidden unless: Question "Does the adult on the spectrum have a history of epilepsy that has been diagnosed by a healthcare professional? ("Epilepsy" can be preceded by but does not include fever-related [febrile] seizures.)" #26 is one of the following answers ("Yes")**

**29) Did the epilepsy begin within weeks or months following a head injury, stroke, serious brain infection requiring hospitalization, or the development of a brain tumor?**

( ) Yes

( ) No

( ) Don't know

( ) Other, please specify:: _______________________________________________

**Logic: Hidden unless: Question "Did the epilepsy begin within weeks or months following a head injury, stroke, serious brain infection requiring hospitalization, or the development of a brain tumor?" #29 is one of the following answers ("Yes")**

## 30) Please describe what occurred in greater detail.

____________________________________________

____________________________________________

____________________________________________

____________________________________________

**Logic: Show/hide trigger exists. Hidden unless: Question "Does the adult on the spectrum have a history of epilepsy that has been diagnosed by a healthcare professional? ("Epilepsy" can be preceded by but does not include fever-related [febrile] seizures.)" #26 is one of the following answers ("Yes")**

**31) If the adult on the spectrum has had a diagnosis of epilepsy, has she been treated with anti-seizure medications?**

( ) Yes

( ) No

( ) Don't know

( ) Other, please explain:: _______________________________________________

**Logic: Hidden unless: Question "If the adult on the spectrum has had a diagnosis of epilepsy, has she been treated with anti-seizure medications?" #31 is one of the following answers ("Yes")**

**32) Is the adult on the spectrum currently on any anti-seizure medications?**

( ) Yes

( ) No

( ) Don't know

( ) Other, please explain:: _________________________________________________

**33) Regardless of whether she has epilepsy, has the adult on the spectrum ever taken any of the following forms of Depakote for an extended period of time (4 or more consecutive months)? Please check all that apply and in the box to the right indicate approximately how long she has been or was on the medication.**

[ ] Depakote: _________________________________________________

[ ] Depakote DR: _________________________________________________

[ ] Depakote ER: _________________________________________________

[ ] Depakene: _________________________________________________

[ ] Stavzor: _________________________________________________

[ ] Valproate: _________________________________________________

[ ] Valproic Acid: _________________________________________________

**34) Regardless of whether she has epilepsy, has the adult on the spectrum ever taken any of the following forms of Tegretol for an extended period of time (4 or more consecutive months)? Please check all that apply and in the box to the right indicate approximately how long she has been or was on the medication.**

[ ] Tegretol: _________________________________________________

[ ] Tegretol-XR: _________________________________________________

[ ] Carbatrol: _________________________________________________

[ ] Epitol: _________________________________________________

[ ] Equetro: _________________________________________________

[ ] Carbamazepine: _________________________________________________

**Logic: Hidden unless: (Question "Regardless of whether she has epilepsy, has the adult on the spectrum ever taken any of the following forms of Tegretol for an extended period of time (4 or more consecutive months)? Please check all that apply and in the box to the right indicate approximately how long she has been or was on the medication." #34 is one of the following answers ("Tegretol","Tegretol-XR","Carbatrol","Epitol","Equetro","Carbamazepine") OR Question "Regardless of whether she has epilepsy, has the adult on the spectrum ever taken any of the following forms of Depakote for an extended period of time (4 or more consecutive months)? Please check all that apply and in the box to the right indicate approximately how long she has been or was on the medication." #33 is one of the following answers ("Depakote","Depakote DR","Depakote ER","Depakene","Stavzor","Valproate","Valproic Acid"))**

**35) If she has taken some form of Depakote and/or Tegretol for an extended period of time, did she take either of them during or after puberty?**

( ) Yes

( ) No

( ) Don't know

( ) Other, please explain:: _______________________________________________

**Logic: Hidden unless: Question "Does the adult on the spectrum have a history of epilepsy that has been diagnosed by a healthcare professional? ("Epilepsy" can be preceded by but does not include fever-related [febrile] seizures.)" #26 is one of the following answers ("Yes")**

**36) Do her seizures worsen in relation to her menstrual cycle?**

( ) No

( ) Yes, they're the worst early in the menstrual cycle (shortly after completing menstruation)

( ) Yes, they're the worst around the middle of the menstrual cycle (around day 14)

( ) Yes, they're the worst closer to or during menstruation

( ) Don't know

( ) Other, please explain:: _______________________________________________

**37) Is there anything else on the topic of epilepsy that you would like to tell us?**

____________________________________________

____________________________________________

____________________________________________

____________________________________________

---

# Endocrine (Hormonal) Conditions:

**This section of the survey addresses issues relating to hormones.**

**38) Does the adult on the spectrum have a diagnosis of Polycystic Ovary Syndrome (PCOS)?**

( ) Yes

( ) Suspected, but not professionally diagnosed

( ) No

( ) Don't know

( ) Other, please explain:: ________________________________________________

**39) Is the adult on the spectrum currently having any hormone treatment, such as birth control pills or any other kind of medication that affects her menstrual cycle? If so, please list the medication(s) below.**

( ) Yes:: ________________________________________________

( ) No

( ) Don't know

( ) Other, please explain:: ________________________________________________

**40) Does the adult on the spectrum have or ever had any of the following? Please check all that apply. (NOTE: Changes to the menstrual cycle should not be the result of conditions such as pregnancy, anorexia, or hormone treatment.)**

[ ] Amenorrhea (one or more missed menstrual periods)

[ ] Diabetes II or insulin resistance (pre-diabetes)

[ ] Endometriosis

[ ] Excessive adult acne

[ ] Female fertility problems

[ ] Frequent dysmenorrhea (severe pain during menstruation)

[ ] Chronic irregular menstrual cycles

[ ] High LDL (bad) cholesterol

[ ] Hypertension (high blood pressure)

[ ] Hirsutism (excessive hair growth, e.g., legs, arms, face)

[ ] Overweight/obese (current or previous)

[ ] Premenstrual Dysphoric Disorder (PMDD) - frequent severe PMS

[ ] Severe acne during puberty

[ ] Uterine fibroids

**Logic: Show/hide trigger exists.**

**41) Has the adult on the spectrum ever been pregnant?**

( ) Yes

( ) No

( ) Don't know

( ) Other, please specify:: ____________________________________________

**Logic: Show/hide trigger exists. Hidden unless: Question "Has the adult on the spectrum ever been pregnant?" #41 is one of the following answers ("Yes")**

**42) Has at least one of those pregnancies been brought to term?**

( ) Yes

( ) No

( ) Don't know

( ) Other, please explain:: ____________________________________________

**Logic: Hidden unless: Question "Has at least one of those pregnancies been brought to term?" #42 is one of the following answers ("Yes")**

**43) Has the adult on the spectrum ever experienced preeclampsia (high blood pressure specifically during pregnancy)?**

( ) Yes

( ) No

( ) Don't know

( ) Other, please specify:: ____________________________________________

**44) Is there anything else about hormone issues the adult may experience that you would like to tell us about?**

____________________________________________

____________________________________________

____________________________________________

____________________________________________

---

## Exercise Habits

**This section addresses exercise habits.**

**Logic: Show/hide trigger exists.**

**45) How often does the adult on the spectrum exercise?**

( ) Daily

( ) Several days a week

( ) Once a week

( ) Infrequently

( ) Rarely or never

( ) Don't know

( ) Other, please explain:: ____________________________________________

**Logic: Hidden unless: Question "How often does the adult on the spectrum exercise?" #45 is one of the following answers ("Daily","Several days a week","Once a week")**

**46) For approximately how long does she exercise during each session?**

( ) 15 minutes

( ) 30 minutes

( ) 45 minutes

( ) 1 hour or more

( ) Too variable to estimate

( ) Don't know

( ) Other, please explain:: ____________________________________________

**Logic: Hidden unless: Question "How often does the adult on the spectrum exercise?" #45 is one of the following answers ("Daily","Several days a week","Once a week")**

**47) When she does exercise, how would you describe the intensity of the exercise?**

( ) Low intensity (e.g., slow-paced walking)

( ) Moderate intensity (e.g., moderate-to-fast-paced walking, yoga)

( ) High intensity (e.g., jogging, intensive weight lifting)

( ) Mixed intensities

( ) Don't know

( ) Other, please explain:: ____________________________________________

**48) Is there anything not covered on the topic of exercise that you would like to tell us?**

____________________________________________

____________________________________________

____________________________________________

____________________________________________

---

# Parents' Cancer History

**This section looks at cancer history in the parents of the adult on the spectrum.**

**Logic: Show/hide trigger exists.**

**49) Do you know some of the medical history of the adult's biological parents?**

( ) Yes

( ) No

**Logic: Show/hide trigger exists. Hidden unless: Question "Do you know some of the medical history of the adult's biological parents?" #49 is one of the following answers ("Yes")**

**50) Is the adult's biological mother alive?**

( ) Yes

( ) No

( ) Don't know

( ) Other, please explain:: _________________________________________________

**Logic: Hidden unless: Question "Is the adult's biological mother alive?" #50 is one of the following answers ("Yes")**

## 51) What age is the adult's biological mother (in years)?

____________________________________________

**Logic: Show/hide trigger exists. Hidden unless: Question "Do you know some of the medical history of the adult's biological parents?" #49 is one of the following answers ("Yes")**

**52) Has the adult's biological mother ever had cancer?**

( ) Yes

( ) No

( ) Don't know

( ) Other, please explain:: ____________________________________________

**Logic: Hidden unless: Question "Has the adult's biological mother ever had cancer?" #52 is one of the following answers ("Yes")**

## 53) At approximately what age did the adult's biological mother initially develop cancer?

____________________________________________

**Logic: Show/hide trigger exists. Hidden unless: Question "Do you know some of the medical history of the adult's biological parents?" #49 is one of the following answers ("Yes")**

**54) Is the adult's biological father alive?**

( ) Yes

( ) No

( ) Don't know

( ) Other, please explain:: ____________________________________________

**Logic: Hidden unless: Question "Is the adult's biological father alive?" #54 is one of the following answers ("Yes")**

### 55) What age is the adult's biological father (in years)?

____________________________________________

**Logic: Show/hide trigger exists. Hidden unless: Question "Do you know some of the medical history of the adult's biological parents?" #49 is one of the following answers ("Yes")**

**56) Has the adult's biological father ever had cancer?**

( ) Yes

( ) No

( ) Don't know

( ) Other, please explain:: ____________________________________________

**Logic: Hidden unless: Question "Has the adult's biological father ever had cancer?" #56 is one of the following answers ("Yes")**

### 57) At approximately what age did the adult's biological father initially develop cancer?

____________________________________________

---

**Logic: Hidden unless: Question "Do you agree to participate in this survey study?" #1 is one of the following answers ("No, I do not consent.")**

**If you meet requirements for inclusion in this study and should decide to participate at a later date, we encourage you to return to this website and complete the survey. Thank you!**

---

# Thank You!

**Thank you! If you have any questions, comments, or concerns, please contact Dr. Emily Casanova at ecasanova@ghs.org or Dr. Manuel Casanova at mcasanova@ghs.org.**

---

**SUPPLEMENTARY TABLES**

**Supplementary Table 1.** Immune symptoms: hospitalization history, asthma, ear infections, rhinitis, and sinusitis. 0 = no symptom reported; 1 = symptom reported.

| ID# | Group | Hospitalization | Asthma | Ear Infections | Rhiniti |
|---|---|---|---|---|---|
| 1 | ASD | 0 | 0 | 0 | 0 |
| 2 | ASD | 0 | 0 | 0 | 0 |
| 3 | ASD | 0 | 0 | 0 | 0 |
| 4 | ASD | 0 | 0 | 0 | 0 |
| 5 | ASD | 1 | 1 | 0 | 0 |
| 6 | ASD | 1 | 1 | 0 | 0 |
| 7 | ASD | 1 | 0 | 0 | 0 |
| 8 | ASD | 0 | 0 | 0 | 1 |
| 9 | ASD | 0 | 1 | 0 | 1 |
| 10 | ASD | 0 | 0 | 0 | 0 |
| 11 | ASD | 0 | 0 | 0 | 0 |
| 12 | ASD | 0 | 1 | 0 | 0 |
| 13 | ASD | 1 | 1 | 0 | 1 |
| 14 | ASD | 1 | 0 | 0 | 0 |
| 15 | ASD | 0 | 1 | 0 | 0 |
| 16 | ASD | 1 | 1 | 1 | 1 |
| 17 | ASD | 1 | 0 | 0 | 0 |
| 18 | ASD | 1 | 1 | 0 | 1 |
| 19 | ASD | 0 | 0 | 1 | 1 |
| 20 | ASD | 0 | 0 | 0 | 0 |
| 21 | ASD | 0 | 1 | 0 | 0 |
| 22 | ASD | 1 | 0 | 0 | 1 |
| 23 | ASD | 1 | 1 | 1 | 0 |
| 24 | ASD | 0 | 0 | 0 | 0 |
| 25 | ASD | 0 | 1 | 0 | 0 |
| 26 | ASD | 1 | 1 | 1 | 0 |
| 27 | ASD | 0 | 1 | 0 | 1 |
| 28 | ASD | 0 | 1 | 1 | 1 |
| 29 | ASD | 0 | 1 | 1 | 1 |
| 30 | ASD | 0 | 0 | 0 | 0 |
| 31 | ASD | 0 | 0 | 1 | 0 |
| 32 | ASD | 0 | 0 | 1 | 1 |
| 33 | ASD | 0 | 0 | 0 | 0 |
| 34 | ASD | 0 | 0 | 1 | 0 |
| 35 | ASD | 0 | 0 | 1 | 1 |
| 36 | ASD | 0 | 0 | 0 | 0 |
| 37 | ASD | 0 | 0 | 1 | 1 |

| | | | | | |
|---|---|---|---|---|---|
| 38 | ASD | 1 | 0 | 1 | 0 |
| 39 | ASD | 0 | 0 | 0 | 0 |
| 40 | ASD | 0 | 0 | 1 | 1 |
| 41 | ASD | 1 | 1 | 1 | 1 |
| 42 | ASD | 0 | 0 | 1 | 0 |
| 43 | ASD | 1 | 1 | 0 | 0 |
| 44 | ASD | 0 | 0 | 0 | 0 |
| 45 | ASD | 1 | 0 | 0 | 0 |
| 46 | ASD | 0 | 0 | 0 | 0 |
| 47 | ASD | 0 | 0 | 1 | 0 |
| 48 | ASD | 0 | 0 | 0 | 0 |
| 49 | ASD | 0 | 0 | 0 | 0 |
| 50 | ASD | 0 | 0 | 0 | 0 |
| 51 | ASD | 0 | 0 | 1 | 1 |
| 52 | ASD | 0 | 0 | 1 | 0 |
| 53 | ASD | 0 | 1 | 0 | 0 |
| 54 | ASD | 0 | 0 | 0 | 1 |
| 55 | ASD | 1 | 1 | 1 | 1 |
| 56 | ASD | 1 | 1 | 0 | 1 |
| 57 | ASD | 0 | 1 | 0 | 0 |
| 58 | ASD | 0 | 0 | 0 | 0 |
| 59 | ASD | 0 | 0 | 0 | 0 |
| 60 | ASD | 0 | 0 | 1 | 1 |
| 61 | ASD | 0 | 1 | 1 | 0 |
| 62 | ASD | 0 | 1 | 1 | 1 |
| 63 | ASD | 0 | 0 | 1 | 1 |
| 64 | ASD | 0 | 0 | 0 | 0 |
| 65 | ASD | 0 | 0 | 0 | 0 |
| 66 | ASD | 0 | 0 | 0 | 1 |
| 67 | ASD | 1 | 1 | 1 | 1 |
| 68 | ASD | 0 | 1 | 1 | 0 |
| 69 | ASD | 0 | 0 | 1 | 1 |
| 70 | ASD | 0 | 0 | 0 | 0 |
| 71 | ASD | 0 | 0 | 0 | 1 |
| 72 | ASD | 1 | 0 | 1 | 0 |
| 73 | ASD | 0 | 1 | 1 | 1 |
| 74 | ASD | 1 | 0 | 0 | 0 |
| 75 | ASD | 1 | 0 | 0 | 1 |
| 76 | ASD | 1 | 0 | 0 | 0 |
| 77 | ASD | 0 | 0 | 0 | 1 |
| 78 | ASD | 0 | 0 | 1 | 0 |

| | | | | | |
|---|---|---|---|---|---|
| 79 | ASD | 1 | 0 | 1 | 1 |
| 80 | ASD | 0 | 1 | 1 | 1 |
| 81 | ASD | 1 | 0 | 1 | 1 |
| 82 | ASD | 0 | 0 | 1 | 0 |
| 83 | ASD | 0 | 1 | 1 | 0 |
| 84 | ASD | 0 | 0 | 0 | 0 |
| 85 | ASD | 0 | 0 | 0 | 0 |
| 86 | ASD/GJH | 0 | 1 | 1 | 1 |
| 87 | ASD/GJH | 1 | 1 | 1 | 1 |
| 88 | ASD/GJH | 0 | 1 | 0 | 1 |
| 89 | ASD/GJH | 0 | 0 | 1 | 1 |
| 90 | ASD/GJH | 1 | 1 | 0 | 0 |
| 91 | ASD/GJH | 0 | 1 | 1 | 1 |
| 92 | ASD/EDS | 1 | 1 | 1 | 1 |
| 93 | ASD/GJH | 1 | 1 | 1 | 1 |
| 94 | ASD/GJH | 0 | 1 | 1 | 1 |
| 95 | ASD/GJH | 1 | 1 | 0 | 0 |
| 96 | ASD/GJH | 0 | 0 | 0 | 0 |
| 97 | ASD/GJH | 1 | 0 | 0 | 0 |
| 98 | ASD/GJH | 1 | 1 | 1 | 1 |
| 99 | ASD/GJH | 0 | 0 | 1 | 0 |
| 100 | ASD/GJH | 0 | 0 | 0 | 1 |
| 101 | ASD/GJH | 1 | 1 | 1 | 1 |
| 102 | ASD/GJH | 1 | 0 | 1 | 0 |
| 103 | ASD/GJH | 0 | 1 | 0 | 0 |
| 104 | ASD/GJH | 1 | 0 | 1 | 0 |
| 105 | ASD/GJH | 0 | 0 | 1 | 1 |

**Supplementary Table 2.** Immune symptoms: allergies, reaction to medications (med react), reaction to environmental chemicals (env react), autoimmunity, and sum of all immune symptoms. 0 = no symptom reported; 1 = symptom reported.

| ID# | Group | Allergies | Med React | Env React | Autoimm |
|---|---|---|---|---|---|
| 1 | ASD | 0 | 0 | 0 | 0 |
| 2 | ASD | 0 | 0 | 0 | 0 |
| 3 | ASD | 0 | 0 | 0 | 0 |
| 4 | ASD | 1 | 0 | 0 | 0 |
| 5 | ASD | 0 | 0 | 0 | 0 |
| 6 | ASD | 0 | 0 | 0 | 0 |
| 7 | ASD | 0 | 0 | 0 | 0 |

| | | | | | |
|---|---|---|---|---|---|
| 8 | ASD | 0 | 1 | 1 | 0 |
| 9 | ASD | 0 | 0 | 1 | 0 |
| 10 | ASD | 0 | 0 | 0 | 0 |
| 11 | ASD | 0 | 0 | 0 | 0 |
| 12 | ASD | 0 | 0 | 0 | 0 |
| 13 | ASD | 1 | 1 | 1 | 0 |
| 14 | ASD | 0 | 0 | 0 | 0 |
| 15 | ASD | 1 | 0 | 1 | 0 |
| 16 | ASD | 1 | 1 | 1 | 1 |
| 17 | ASD | 0 | 0 | 0 | 0 |
| 18 | ASD | 0 | 0 | 0 | 0 |
| 19 | ASD | 0 | 0 | 1 | 0 |
| 20 | ASD | 0 | 1 | 1 | 0 |
| 21 | ASD | 0 | 0 | 0 | 1 |
| 22 | ASD | 1 | 0 | 1 | 0 |
| 23 | ASD | 1 | 1 | 1 | 0 |
| 24 | ASD | 0 | 0 | 0 | 0 |
| 25 | ASD | 0 | 0 | 0 | 0 |
| 26 | ASD | 1 | 0 | 0 | 1 |
| 27 | ASD | 1 | 1 | 1 | 0 |
| 28 | ASD | 1 | 0 | 0 | 1 |
| 29 | ASD | 1 | 1 | 1 | 0 |
| 30 | ASD | 0 | 1 | 1 | 0 |
| 31 | ASD | 0 | 0 | 0 | 0 |
| 32 | ASD | 0 | 0 | 0 | 0 |
| 33 | ASD | 0 | 0 | 0 | 0 |
| 34 | ASD | 0 | 0 | 0 | 1 |
| 35 | ASD | 1 | 1 | 1 | 0 |
| 36 | ASD | 0 | 1 | 0 | 0 |
| 37 | ASD | 0 | 0 | 1 | 0 |
| 38 | ASD | 1 | 1 | 1 | 0 |
| 39 | ASD | 0 | 0 | 1 | 0 |
| 40 | ASD | 1 | 0 | 0 | 0 |
| 41 | ASD | 1 | 1 | 1 | 0 |
| 42 | ASD | 1 | 1 | 0 | 0 |
| 43 | ASD | 1 | 0 | 1 | 0 |
| 44 | ASD | 0 | 0 | 0 | 0 |
| 45 | ASD | 0 | 1 | 0 | 0 |
| 46 | ASD | 0 | 0 | 0 | 0 |
| 47 | ASD | 0 | 0 | 0 | 0 |
| 48 | ASD | 0 | 0 | 0 | 0 |

| | | | | | |
|---|---|---|---|---|---|
| 49 | ASD | 0 | 0 | 0 | 0 |
| 50 | ASD | 1 | 0 | 0 | 0 |
| 51 | ASD | 0 | 0 | 0 | 0 |
| 52 | ASD | 0 | 0 | 0 | 0 |
| 53 | ASD | 0 | 0 | 1 | 0 |
| 54 | ASD | 1 | 0 | 0 | 0 |
| 55 | ASD | 1 | 1 | 1 | 0 |
| 56 | ASD | 1 | 1 | 1 | 0 |
| 57 | ASD | 0 | 0 | 0 | 1 |
| 58 | ASD | 0 | 1 | 0 | 0 |
| 59 | ASD | 1 | 0 | 0 | 0 |
| 60 | ASD | 1 | 0 | 1 | 0 |
| 61 | ASD | 0 | 1 | 1 | 0 |
| 62 | ASD | 1 | 1 | 1 | 0 |
| 63 | ASD | 0 | 1 | 0 | 0 |
| 64 | ASD | 1 | 1 | 1 | 0 |
| 65 | ASD | 0 | 0 | 0 | 0 |
| 66 | ASD | 1 | 0 | 0 | 0 |
| 67 | ASD | 1 | 0 | 0 | 1 |
| 68 | ASD | 1 | 1 | 0 | 0 |
| 69 | ASD | 1 | 1 | 1 | 0 |
| 70 | ASD | 0 | 0 | 0 | 1 |
| 71 | ASD | 1 | 0 | 1 | 0 |
| 72 | ASD | 1 | 0 | 0 | 0 |
| 73 | ASD | 1 | 1 | 0 | 1 |
| 74 | ASD | 1 | 0 | 1 | 1 |
| 75 | ASD | 1 | 1 | 1 | 0 |
| 76 | ASD | 1 | 1 | 1 | 0 |
| 77 | ASD | 0 | 0 | 0 | 0 |
| 78 | ASD | 0 | 0 | 0 | 0 |
| 79 | ASD | 1 | 1 | 1 | 1 |
| 80 | ASD | 1 | 1 | 1 | 0 |
| 81 | ASD | 1 | 1 | 0 | 0 |
| 82 | ASD | 0 | 0 | 0 | 0 |
| 83 | ASD | 0 | 0 | 1 | 0 |
| 84 | ASD | 1 | 0 | 0 | 0 |
| 85 | ASD | 0 | 1 | 0 | 0 |
| 86 | ASD/GJH | 0 | 1 | 1 | 0 |
| 87 | ASD/GJH | 1 | 1 | 1 | 1 |
| 88 | ASD/GJH | 1 | 0 | 1 | 0 |
| 89 | ASD/GJH | 0 | 1 | 0 | 1 |

| | | | | | |
|---|---|---|---|---|---|
| 90 | ASD/GJH | 1 | 0 | 1 | 0 |
| 91 | ASD/GJH | 1 | 0 | 0 | 0 |
| 92 | ASD/GJH | 1 | 1 | 1 | 0 |
| 93 | ASD/GJH | 0 | 0 | 0 | 1 |
| 94 | ASD/GJH | 1 | 1 | 1 | 1 |
| 95 | ASD/GJH | 0 | 1 | 1 | 0 |
| 96 | ASD/GJH | 1 | 1 | 0 | 1 |
| 97 | ASD/GJH | 0 | 1 | 1 | 0 |
| 98 | ASD/GJH | 1 | 1 | 1 | 1 |
| 99 | ASD/GJH | 1 | 1 | 1 | 0 |
| 100 | ASD/GJH | 1 | 1 | 1 | 0 |
| 101 | ASD/GJH | 1 | 1 | 1 | 1 |
| 102 | ASD/GJH | 0 | 0 | 0 | 1 |
| 103 | ASD/GJH | 1 | 0 | 0 | 0 |
| 104 | ASD/GJH | 0 | 1 | 1 | 1 |
| 105 | ASD/GJH | 0 | 0 | 0 | 0 |

**Supplementary Table 3.** Endocrine symptoms: polycystic ovary syndrome (PCOS), amenorrhea, diabetes 2/insulin resistance (diabetes, endometriosis, and adult acne. 0 = no symptom reported; 1 = symptom reported.

| ID# | Group | PCOS | Amenorrhea | Diabetes | Endometri |
|---|---|---|---|---|---|
| 1 | ASD | 0 | 0 | 0 | 0 |
| 2 | ASD | 0 | 0 | 0 | 0 |
| 3 | ASD | 0 | 0 | 0 | 0 |
| 4 | ASD | 0 | 0 | 0 | 0 |
| 5 | ASD | 0 | 0 | 0 | 0 |
| 6 | ASD | 0 | 1 | 0 | 0 |
| 7 | ASD | 0 | 0 | 0 | 0 |
| 8 | ASD | 0 | 0 | 0 | 0 |
| 9 | ASD | 0 | 0 | 1 | 0 |
| 10 | ASD | 0 | 0 | 0 | 0 |
| 11 | ASD | 0 | 0 | 0 | 0 |
| 12 | ASD | 0 | 1 | 0 | 0 |
| 13 | ASD | 0 | 0 | 0 | 0 |
| 14 | ASD | 0 | 1 | 0 | 0 |
| 15 | ASD | 0 | 1 | 0 | 0 |
| 16 | ASD | 0 | 1 | 0 | 0 |
| 17 | ASD | 0 | 0 | 0 | 0 |
| 18 | ASD | 0 | 1 | 0 | 0 |
| 19 | ASD | 0 | 0 | 1 | 0 |
| 20 | ASD | 0 | 1 | 0 | 0 |
| 21 | ASD | 0 | 0 | 0 | 0 |

| | | | | | |
|---|---|---|---|---|---|
| 22 | ASD | 0 | 1 | 0 | 0 |
| 23 | ASD | 0 | 0 | 0 | 0 |
| 24 | ASD | 0 | 1 | 0 | 0 |
| 25 | ASD | 0 | 0 | 0 | 0 |
| 26 | ASD | 0 | 1 | 0 | 0 |
| 27 | ASD | 0 | 0 | 0 | 0 |
| 28 | ASD | 1 | 1 | 1 | 0 |
| 29 | ASD | 0 | 1 | 0 | 0 |
| 30 | ASD | 0 | 1 | 0 | 0 |
| 31 | ASD | 0 | 1 | 0 | 0 |
| 32 | ASD | 0 | 0 | 0 | 0 |
| 33 | ASD | 0 | 0 | 0 | 0 |
| 34 | ASD | 0 | 0 | 0 | 0 |
| 35 | ASD | 1 | 0 | 0 | 1 |
| 36 | ASD | 0 | 0 | 0 | 0 |
| 37 | ASD | 0 | 0 | 0 | 0 |
| 38 | ASD | 0 | 0 | 0 | 1 |
| 39 | ASD | 0 | 1 | 0 | 0 |
| 40 | ASD | 0 | 0 | 0 | 0 |
| 41 | ASD | 0 | 1 | 0 | 0 |
| 42 | ASD | 0 | 0 | 0 | 0 |
| 43 | ASD | 0 | 1 | 0 | 0 |
| 44 | ASD | 0 | 0 | 0 | 0 |
| 45 | ASD | 0 | 0 | 0 | 0 |
| 46 | ASD | 0 | 0 | 0 | 0 |
| 47 | ASD | 0 | 0 | 0 | 0 |
| 48 | ASD | 0 | 0 | 0 | 0 |
| 49 | ASD | 0 | 0 | 0 | 0 |
| 50 | ASD | 0 | 0 | 0 | 0 |
| 51 | ASD | 0 | 0 | 0 | 0 |
| 52 | ASD | 0 | 0 | 0 | 0 |
| 53 | ASD | 0 | 0 | 0 | 0 |
| 54 | ASD | 0 | 0 | 0 | 0 |
| 55 | ASD | 1 | 1 | 1 | 0 |
| 56 | ASD | 0 | 0 | 0 | 0 |
| 57 | ASD | 0 | 0 | 0 | 0 |
| 58 | ASD | 1 | 1 | 1 | 0 |
| 59 | ASD | 0 | 1 | 0 | 0 |
| 60 | ASD | 0 | 1 | 0 | 1 |
| 61 | ASD | 0 | 1 | 0 | 0 |
| 62 | ASD | 0 | 0 | 0 | 0 |

| | | | | | |
|---|---|---|---|---|---|
| 63 | ASD | 0 | 0 | 0 | 0 |
| 64 | ASD | 0 | 1 | 0 | 0 |
| 65 | ASD | 0 | 1 | 0 | 0 |
| 66 | ASD | 0 | 0 | 0 | 0 |
| 67 | ASD | 0 | 1 | 0 | 0 |
| 68 | ASD | 1 | 1 | 0 | 0 |
| 69 | ASD | 1 | 0 | 0 | 0 |
| 70 | ASD | 0 | 0 | 0 | 0 |
| 71 | ASD | 0 | 0 | 0 | 0 |
| 72 | ASD | 0 | 1 | 0 | 0 |
| 73 | ASD | 0 | 0 | 0 | 0 |
| 74 | ASD | 0 | 0 | 0 | 0 |
| 75 | ASD | 0 | 1 | 0 | 0 |
| 76 | ASD | 0 | 1 | 0 | 1 |
| 77 | ASD | 0 | 0 | 0 | 0 |
| 78 | ASD | 0 | 1 | 0 | 0 |
| 79 | ASD | 0 | 1 | 0 | 0 |
| 80 | ASD | 0 | 0 | 0 | 0 |
| 81 | ASD | 0 | 0 | 0 | 0 |
| 82 | ASD | 0 | 0 | 0 | 0 |
| 83 | ASD | 0 | 1 | 0 | 0 |
| 84 | ASD | 1 | 1 | 0 | 0 |
| 85 | ASD | 0 | 0 | 0 | 0 |
| 86 | ASD/GJH | 0 | 1 | 0 | 0 |
| 87 | ASD/GJH | 0 | 1 | 0 | 0 |
| 88 | ASD/GJH | 0 | 1 | 0 | 0 |
| 89 | ASD/GJH | 0 | 0 | 0 | 1 |
| 90 | ASD/GJH | 0 | 0 | 0 | 0 |
| 91 | ASD/GJH | 0 | 0 | 0 | 0 |
| 92 | ASD/GJH | 0 | 0 | 0 | 0 |
| 93 | ASD/GJH | 1 | 1 | 0 | 0 |
| 94 | ASD/GJH | 0 | 1 | 1 | 1 |
| 95 | ASD/GJH | 0 | 0 | 0 | 0 |
| 96 | ASD/GJH | 0 | 1 | 0 | 0 |
| 97 | ASD/GJH | 0 | 1 | 0 | 1 |
| 98 | ASD/GJH | 1 | 1 | 0 | 1 |
| 99 | ASD/GJH | 0 | 0 | 0 | 0 |
| 100 | ASD/GJH | 0 | 0 | 0 | 0 |
| 101 | ASD/GJH | 1 | 1 | 0 | 1 |
| 102 | ASD/GJH | 0 | 0 | 0 | 0 |
| 103 | ASD/GJH | 0 | 0 | 0 | 1 |

| | | | | | |
|---|---|---|---|---|---|
| 104 | ASD/GJH | 1 | 0 | 0 | 0 |
| 105 | ASD/GJH | 1 | 0 | 1 | 0 |

**Supplementary File 4.** Endocrine symptoms: infertility, dysmenorrhea, irregular menses, high LDL cholesterol, and hypertension. 0 = no symptom reported; 1 = symptom reported.

| ID# | Group | Infertility | Dysmeno-rrhea | Irreg. Menses | High LD |
|---|---|---|---|---|---|
| 1 | ASD | 0 | 0 | 0 | 0 |
| 2 | ASD | 0 | 0 | 0 | 0 |
| 3 | ASD | 1 | 0 | 0 | 0 |
| 4 | ASD | 0 | 0 | 0 | 0 |
| 5 | ASD | 0 | 0 | 0 | 0 |
| 6 | ASD | 1 | 1 | 0 | 0 |
| 7 | ASD | 0 | 1 | 0 | 0 |
| 8 | ASD | 0 | 0 | 0 | 0 |
| 9 | ASD | 0 | 0 | 0 | 1 |
| 10 | ASD | 0 | 0 | 0 | 0 |
| 11 | ASD | 0 | 0 | 0 | 0 |
| 12 | ASD | 0 | 0 | 0 | 0 |
| 13 | ASD | 0 | 0 | 0 | 0 |
| 14 | ASD | 0 | 0 | 0 | 0 |
| 15 | ASD | 0 | 0 | 0 | 0 |
| 16 | ASD | 0 | 0 | 1 | 1 |
| 17 | ASD | 0 | 0 | 0 | 0 |
| 18 | ASD | 0 | 1 | 1 | 1 |
| 19 | ASD | 0 | 0 | 0 | 0 |
| 20 | ASD | 0 | 1 | 1 | 0 |
| 21 | ASD | 0 | 0 | 0 | 0 |
| 22 | ASD | 0 | 0 | 0 | 0 |
| 23 | ASD | 0 | 1 | 0 | 0 |
| 24 | ASD | 0 | 0 | 0 | 0 |
| 25 | ASD | 0 | 1 | 0 | 0 |
| 26 | ASD | 0 | 0 | 1 | 0 |
| 27 | ASD | 0 | 1 | 0 | 1 |
| 28 | ASD | 0 | 0 | 1 | 1 |
| 29 | ASD | 0 | 1 | 1 | 1 |
| 30 | ASD | 0 | 0 | 1 | 0 |
| 31 | ASD | 0 | 0 | 1 | 0 |
| 32 | ASD | 0 | 0 | 0 | 0 |
| 33 | ASD | 0 | 0 | 0 | 0 |
| 34 | ASD | 0 | 1 | 0 | 0 |

| | | | | | |
|---|---|---|---|---|---|
| 35 | ASD | 1 | 0 | 0 | 0 |
| 36 | ASD | 0 | 1 | 0 | 0 |
| 37 | ASD | 0 | 0 | 0 | 0 |
| 38 | ASD | 0 | 1 | 0 | 0 |
| 39 | ASD | 0 | 0 | 0 | 1 |
| 40 | ASD | 0 | 0 | 0 | 0 |
| 41 | ASD | 0 | 1 | 1 | 0 |
| 42 | ASD | 0 | 1 | 0 | 0 |
| 43 | ASD | 0 | 1 | 0 | 0 |
| 44 | ASD | 0 | 1 | 0 | 0 |
| 45 | ASD | 0 | 0 | 0 | 1 |
| 46 | ASD | 0 | 0 | 0 | 0 |
| 47 | ASD | 0 | 0 | 0 | 0 |
| 48 | ASD | 0 | 0 | 0 | 0 |
| 49 | ASD | 0 | 0 | 0 | 0 |
| 50 | ASD | 0 | 1 | 1 | 0 |
| 51 | ASD | 0 | 0 | 0 | 0 |
| 52 | ASD | 0 | 0 | 0 | 0 |
| 53 | ASD | 0 | 0 | 0 | 0 |
| 54 | ASD | 0 | 1 | 0 | 0 |
| 55 | ASD | 1 | 0 | 1 | 1 |
| 56 | ASD | 0 | 0 | 1 | 0 |
| 57 | ASD | 0 | 0 | 0 | 0 |
| 58 | ASD | 0 | 0 | 1 | 0 |
| 59 | ASD | 0 | 0 | 0 | 0 |
| 60 | ASD | 0 | 1 | 1 | 0 |
| 61 | ASD | 0 | 0 | 0 | 0 |
| 62 | ASD | 0 | 0 | 0 | 0 |
| 63 | ASD | 0 | 1 | 1 | 0 |
| 64 | ASD | 0 | 0 | 0 | 0 |
| 65 | ASD | 0 | 1 | 1 | 0 |
| 66 | ASD | 1 | 0 | 0 | 0 |
| 67 | ASD | 0 | 0 | 1 | 0 |
| 68 | ASD | 0 | 0 | 1 | 1 |
| 69 | ASD | 1 | 0 | 0 | 0 |
| 70 | ASD | 0 | 0 | 0 | 0 |
| 71 | ASD | 0 | 0 | 0 | 0 |
| 72 | ASD | 0 | 0 | 0 | 0 |
| 73 | ASD | 0 | 0 | 0 | 1 |
| 74 | ASD | 0 | 1 | 0 | 0 |
| 75 | ASD | 0 | 0 | 0 | 0 |

| | | | | | |
|---|---|---|---|---|---|
| 76 | ASD | 0 | 1 | 1 | 0 |
| 77 | ASD | 0 | 0 | 0 | 1 |
| 78 | ASD | 0 | 0 | 1 | 0 |
| 79 | ASD | 0 | 0 | 1 | 0 |
| 80 | ASD | 0 | 0 | 0 | 0 |
| 81 | ASD | 0 | 0 | 0 | 0 |
| 82 | ASD | 0 | 0 | 0 | 0 |
| 83 | ASD | 0 | 0 | 1 | 0 |
| 84 | ASD | 1 | 1 | 1 | 0 |
| 85 | ASD | 0 | 1 | 0 | 0 |
| 86 | ASD/GJH | 1 | 1 | 1 | 0 |
| 87 | ASD/GJH | 0 | 1 | 0 | 0 |
| 88 | ASD/GJH | 0 | 0 | 1 | 0 |
| 89 | ASD/GJH | 0 | 1 | 0 | 0 |
| 90 | ASD/GJH | 0 | 0 | 0 | 0 |
| 91 | ASD/GJH | 0 | 1 | 0 | 1 |
| 92 | ASD/GJH | 0 | 1 | 0 | 0 |
| 93 | ASD/GJH | 0 | 1 | 1 | 0 |
| 94 | ASD/GJH | 0 | 1 | 1 | 1 |
| 95 | ASD/GJH | 0 | 1 | 0 | 1 |
| 96 | ASD/GJH | 0 | 1 | 1 | 0 |
| 97 | ASD/GJH | 0 | 1 | 1 | 1 |
| 98 | ASD/GJH | 0 | 1 | 1 | 0 |
| 99 | ASD/GJH | 1 | 0 | 0 | 0 |
| 100 | ASD/GJH | 0 | 1 | 1 | 1 |
| 101 | ASD/GJH | 0 | 1 | 1 | 0 |
| 102 | ASD/GJH | 0 | 1 | 0 | 0 |
| 103 | ASD/GJH | 1 | 1 | 0 | 0 |
| 104 | ASD/GJH | 0 | 1 | 1 | 0 |
| 105 | ASD/GJH | 0 | 1 | 1 | 1 |

**Supplementary Table 5.** Endocrine symptoms: hirsutism, overweight/obesity, premenstrual dysphoric disorder (PMDD), severe teen acne, uterine fibroids, and sum of all endocrine symptoms. 0 = no symptom reported; 1 = symptom reported.

| ID# | Group | Hirsutism | Overweight/ Obesity | PMDD | Severe Teen Acne | Ut Fib |
|---|---|---|---|---|---|---|
| 1 | ASD | 0 | 0 | 1 | 0 | |
| 2 | ASD | 0 | 0 | 0 | 0 | |
| 3 | ASD | 0 | 0 | 0 | 0 | |
| 4 | ASD | 0 | 0 | 0 | 0 | |
| 5 | ASD | 1 | 0 | 0 | 0 | |

| | | | | | | |
|---|---|---|---|---|---|---|
| 6 | ASD | 0 | 0 | 0 | 0 | |
| 7 | ASD | 0 | 1 | 1 | 1 | |
| 8 | ASD | 0 | 0 | 0 | 0 | |
| 9 | ASD | 1 | 1 | 0 | 0 | |
| 10 | ASD | 0 | 0 | 0 | 0 | |
| 11 | ASD | 0 | 0 | 0 | 0 | |
| 12 | ASD | 0 | 0 | 0 | 0 | |
| 13 | ASD | 0 | 0 | 0 | 0 | |
| 14 | ASD | 0 | 0 | 1 | 0 | |
| 15 | ASD | 0 | 1 | 0 | 0 | |
| 16 | ASD | 0 | 1 | 1 | 1 | |
| 17 | ASD | 0 | 0 | 0 | 0 | |
| 18 | ASD | 0 | 1 | 0 | 0 | |
| 19 | ASD | 0 | 0 | 0 | 0 | |
| 20 | ASD | 0 | 0 | 0 | 0 | |
| 21 | ASD | 0 | 1 | 0 | 0 | |
| 22 | ASD | 0 | 0 | 0 | 0 | |
| 23 | ASD | 0 | 0 | 1 | 0 | |
| 24 | ASD | 0 | 0 | 0 | 0 | |
| 25 | ASD | 0 | 1 | 0 | 0 | |
| 26 | ASD | 1 | 0 | 1 | 1 | |
| 27 | ASD | 0 | 1 | 0 | 0 | |
| 28 | ASD | 1 | 1 | 0 | 0 | |
| 29 | ASD | 0 | 0 | 0 | 0 | |
| 30 | ASD | 0 | 0 | 1 | 1 | |
| 31 | ASD | 1 | 1 | 0 | 0 | |
| 32 | ASD | 0 | 0 | 0 | 0 | |
| 33 | ASD | 0 | 0 | 1 | 1 | |
| 34 | ASD | 0 | 0 | 0 | 0 | |
| 35 | ASD | 0 | 0 | 0 | 0 | |
| 36 | ASD | 0 | 1 | 0 | 0 | |
| 37 | ASD | 1 | 1 | 0 | 0 | |
| 38 | ASD | 0 | 0 | 0 | 0 | |
| 39 | ASD | 0 | 0 | 0 | 0 | |
| 40 | ASD | 0 | 0 | 0 | 0 | |
| 41 | ASD | 0 | 0 | 1 | 0 | |
| 42 | ASD | 1 | 0 | 0 | 0 | |
| 43 | ASD | 0 | 1 | 0 | 0 | |
| 44 | ASD | 0 | 1 | 0 | 0 | |
| 45 | ASD | 0 | 1 | 0 | 0 | |
| 46 | ASD | 0 | 1 | 1 | 0 | |

| | | | | | | |
|---|---|---|---|---|---|---|
| 47 | ASD | 0 | 0 | 0 | 0 | |
| 48 | ASD | 0 | 0 | 0 | 0 | |
| 49 | ASD | 0 | 0 | 0 | 0 | |
| 50 | ASD | 1 | 0 | 0 | 1 | |
| 51 | ASD | 0 | 0 | 0 | 0 | |
| 52 | ASD | 1 | 0 | 0 | 1 | |
| 53 | ASD | 0 | 0 | 0 | 0 | |
| 54 | ASD | 0 | 0 | 0 | 0 | |
| 55 | ASD | 0 | 1 | 1 | 0 | |
| 56 | ASD | 0 | 0 | 1 | 0 | |
| 57 | ASD | 0 | 1 | 0 | 0 | |
| 58 | ASD | 1 | 0 | 0 | 1 | |
| 59 | ASD | 0 | 0 | 0 | 0 | |
| 60 | ASD | 0 | 0 | 0 | 1 | |
| 61 | ASD | 0 | 0 | 0 | 0 | |
| 62 | ASD | 0 | 0 | 1 | 0 | |
| 63 | ASD | 0 | 0 | 0 | 0 | |
| 64 | ASD | 0 | 0 | 0 | 0 | |
| 65 | ASD | 0 | 1 | 0 | 0 | |
| 66 | ASD | 1 | 1 | 0 | 0 | |
| 67 | ASD | 0 | 1 | 0 | 1 | |
| 68 | ASD | 1 | 0 | 1 | 1 | |
| 69 | ASD | 0 | 0 | 0 | 0 | |
| 70 | ASD | 0 | 1 | 0 | 0 | |
| 71 | ASD | 0 | 1 | 0 | 0 | |
| 72 | ASD | 1 | 0 | 0 | 0 | |
| 73 | ASD | 0 | 0 | 0 | 0 | |
| 74 | ASD | 0 | 0 | 1 | 0 | |
| 75 | ASD | 0 | 1 | 1 | 0 | |
| 76 | ASD | 1 | 1 | 1 | 0 | |
| 77 | ASD | 1 | 1 | 0 | 1 | |
| 78 | ASD | 1 | 1 | 0 | 0 | |
| 79 | ASD | 0 | 1 | 0 | 0 | |
| 80 | ASD | 0 | 1 | 0 | 0 | |
| 81 | ASD | 0 | 0 | 0 | 0 | |
| 82 | ASD | 0 | 1 | 0 | 0 | |
| 83 | ASD | 0 | 1 | 0 | 0 | |
| 84 | ASD | 0 | 0 | 1 | 0 | |
| 85 | ASD | 0 | 0 | 0 | 0 | |
| 86 | ASD/GJH | 0 | 0 | 0 | 1 | |
| 87 | ASD/GJH | 0 | 1 | 1 | 0 | |

| | | | | | |
|---|---|---|---|---|---|
| 88 | ASD/GJH | 1 | 1 | 0 | 0 |
| 89 | ASD/GJH | 0 | 0 | 0 | 1 |
| 90 | ASD/GJH | 0 | 0 | 0 | 0 |
| 91 | ASD/GJH | 0 | 0 | 0 | 0 |
| 92 | ASD/GJH | 0 | 0 | 0 | 0 |
| 93 | ASD/GJH | 0 | 1 | 1 | 1 |
| 94 | ASD/GJH | 0 | 0 | 1 | 1 |
| 95 | ASD/GJH | 0 | 0 | 1 | 0 |
| 96 | ASD/GJH | 1 | 1 | 1 | 0 |
| 97 | ASD/GJH | 0 | 1 | 0 | 1 |
| 98 | ASD/GJH | 1 | 0 | 0 | 1 |
| 99 | ASD/GJH | 0 | 0 | 0 | 0 |
| 100 | ASD/GJH | 0 | 1 | 0 | 0 |
| 101 | ASD/GJH | 1 | 1 | 0 | 1 |
| 102 | ASD/GJH | 0 | 0 | 0 | 1 |
| 103 | ASD/GJH | 0 | 0 | 0 | 0 |
| 104 | ASD/GJH | 1 | 1 | 1 | 1 |
| 105 | ASD/GJH | 1 | 1 | 0 | 0 |

**Supplementary Table 6.** Sum of immune symptoms by age range (child, teen, and adult). 0 = no symptom reported; 1 = symptom reported.

| ID# | Group | Child Immune | Teen Immune |
|---|---|---|---|
| 1 | ASD | 0 | 0 |
| 2 | ASD | 0 | 1 |
| 3 | ASD | 0 | 0 |
| 4 | ASD | 0 | 1 |
| 5 | ASD | 2 | 1 |
| 6 | ASD | 1 | 0 |
| 7 | ASD | 1 | 0 |
| 8 | ASD | 3 | 2 |
| 9 | ASD | 3 | 2 |
| 10 | ASD | 0 | 1 |
| 11 | ASD | 0 | 0 |
| 12 | ASD | 1 | 1 |
| 13 | ASD | 0 | 1 |
| 14 | ASD | 1 | 0 |
| 15 | ASD | 2 | 3 |
| 16 | ASD | 4 | 5 |
| 17 | ASD | 1 | 0 |
| 18 | ASD | 1 | 2 |
| 19 | ASD | 2 | 2 |

| | | | | |
|---|---|---|---|---|
| 20 | ASD | 3 | 3 | |
| 21 | ASD | 1 | 1 | |
| 22 | ASD | 2 | 3 | |
| 23 | ASD | 6 | 3 | |
| 24 | ASD | 0 | 0 | |
| 25 | ASD | 0 | 0 | |
| 26 | ASD | 4 | 2 | |
| 27 | ASD | 1 | 1 | |
| 28 | ASD | 4 | 4 | |
| 29 | ASD | 5 | 6 | |
| 30 | ASD | 0 | 2 | |
| 31 | ASD | 1 | 0 | |
| 32 | ASD | 2 | 2 | |
| 33 | ASD | 0 | 0 | |
| 34 | ASD | 1 | 0 | |
| 35 | ASD | 3 | 4 | |
| 36 | ASD | 1 | 0 | |
| 37 | ASD | 1 | 2 | |
| 38 | ASD | 2 | 4 | |
| 39 | ASD | 0 | 0 | |
| 40 | ASD | 1 | 0 | |
| 41 | ASD | 2 | 7 | |
| 42 | ASD | 1 | 0 | |
| 43 | ASD | 4 | 0 | |
| 44 | ASD | 0 | 0 | |
| 45 | ASD | 0 | 1 | |
| 46 | ASD | 0 | 0 | |
| 47 | ASD | 2 | 1 | |
| 48 | ASD | 0 | 0 | |
| 49 | ASD | 0 | 0 | |
| 50 | ASD | 1 | 1 | |
| 51 | ASD | 2 | 2 | |
| 52 | ASD | 0 | 1 | |
| 53 | ASD | 0 | 2 | |
| 54 | ASD | 0 | 0 | |
| 55 | ASD | 6 | 5 | |
| 56 | ASD | 5 | 5 | |
| 57 | ASD | 1 | 0 | |
| 58 | ASD | 0 | 1 | |
| 59 | ASD | 1 | 2 | |
| 60 | ASD | 5 | 5 | |

| | | | | |
|---|---|---|---|---|
| 61 | ASD | 1 | 0 | |
| 62 | ASD | 5 | 5 | |
| 63 | ASD | 3 | 3 | |
| 64 | ASD | 3 | 4 | |
| 65 | ASD | 1 | 1 | |
| 66 | ASD | 0 | 1 | |
| 67 | ASD | 6 | 5 | |
| 68 | ASD | 5 | 4 | |
| 69 | ASD | 6 | 6 | |
| 70 | ASD | 0 | 0 | |
| 71 | ASD | 4 | 4 | |
| 72 | ASD | 2 | 2 | |
| 73 | ASD | 3 | 3 | |
| 74 | ASD | 3 | 2 | |
| 75 | ASD | 5 | 5 | |
| 76 | ASD | 1 | 1 | |
| 77 | ASD | 0 | 0 | |
| 78 | ASD | 1 | 0 | |
| 79 | ASD | 5 | 7 | |
| 80 | ASD | 2 | 5 | |
| 81 | ASD | 6 | 5 | |
| 82 | ASD | 1 | 0 | |
| 83 | ASD | 1 | 0 | |
| 84 | ASD | 0 | 0 | |
| 85 | ASD | 0 | 1 | |
| 86 | ASD/GJH | 2 | 3 | |
| 87 | ASD/GJH | 5 | 7 | |
| 88 | ASD/GJH | 4 | 3 | |
| 89 | ASD/GJH | 3 | 3 | |
| 90 | ASD/GJH | 4 | 3 | |
| 91 | ASD/GJH | 1 | 3 | |
| 92 | ASD/GJH | 7 | 4 | |
| 93 | ASD/GJH | 3 | 3 | |
| 94 | ASD/GJH | 5 | 6 | |
| 95 | ASD/GJH | 1 | 1 | |
| 96 | ASD/GJH | 0 | 0 | |
| 97 | ASD/GJH | 1 | 0 | |
| 98 | ASD/GJH | 3 | 2 | |
| 99 | ASD/GJH | 5 | 4 | |
| 100 | ASD/GJH | 3 | 3 | |
| 101 | ASD/GJH | 5 | 6 | |

| | | | |
|---|---|---|---|
| 102 | ASD/GJH | 1 | 1 |
| 103 | ASD/GJH | 0 | 0 |
| 104 | ASD/GJH | 2 | 2 |
| 105 | ASD/GJH | 3 | 0 |

**Supplementary Table 7.** Symptoms of irritable bowel syndrome/gastrointestinal dysmotility (IBS), joint pain, other chronic pain, epilepsy, and birth control/hormone treatment (BC Tx). 0 = no symptom reported; 1 = symptom reported.

| ID# | Group | IBS | Joint Pain | Other Chronic Pain | Epileps |
|---|---|---|---|---|---|
| 1 | ASD | 0 | 0 | 2 | 0 |
| 2 | ASD | 0 | 0 | 2 | 0 |
| 3 | ASD | 1 | 0 | 2 | 0 |
| 4 | ASD | 0 | 0 | 2 | 0 |
| 5 | ASD | 1 | 0 | 2 | 0 |
| 6 | ASD | 1 | 1 | 2 | 0 |
| 7 | ASD | 1 | 1 | 2 | 0 |
| 8 | ASD | 0 | 0 | 1 | 0 |
| 9 | ASD | 1 | 1 | 2 | 0 |
| 10 | ASD | 0 | 0 | 2 | 0 |
| 11 | ASD | 0 | 1 | 2 | 0 |
| 12 | ASD | 0 | 0 | 2 | 0 |
| 13 | ASD | 0 | 1 | 2 | 1 |
| 14 | ASD | 1 | 0 | 3 | 0 |
| 15 | ASD | 1 | 0 | 2 | 0 |
| 16 | ASD | 1 | 1 | 1 | 0 |
| 17 | ASD | 1 | 0 | 2 | 0 |
| 18 | ASD | 1 | 1 | 1 | 0 |
| 19 | ASD | 1 | 1 | 1 | 0 |
| 20 | ASD | 1 | 1 | 4 | 0 |
| 21 | ASD | 0 | 0 | 2 | 0 |
| 22 | ASD | 1 | 0 | 2 | 0 |
| 23 | ASD | 1 | 0 | 2 | 0 |
| 24 | ASD | 1 | 0 | 2 | 0 |
| 25 | ASD | 1 | 0 | 2 | 0 |
| 26 | ASD | 0 | 1 | 3 | 0 |
| 27 | ASD | 1 | 1 | 1 | 0 |
| 28 | ASD | 1 | 0 | 1 | 1 |
| 29 | ASD | 0 | 0 | 2 | 0 |
| 30 | ASD | 1 | 0 | 4 | 0 |
| 31 | ASD | 1 | 0 | 4 | 1 |
| 32 | ASD | 0 | 0 | no response | 0 |

| | | | | | |
|---|---|---|---|---|---|
| 33 | ASD | 1 | 0 | 2 | 0 |
| 34 | ASD | 1 | 0 | 2 | 0 |
| 35 | ASD | 1 | 0 | 2 | 0 |
| 36 | ASD | 0 | 0 | 2 | 0 |
| 37 | ASD | 0 | 0 | 2 | 0 |
| 38 | ASD | 1 | 0 | 1 | 1 |
| 39 | ASD | 1 | 0 | 1 | 0 |
| 40 | ASD | 1 | 0 | 2 | 0 |
| 41 | ASD | 0 | 1 | 1 | 0 |
| 42 | ASD | 1 | 0 | 1 | 0 |
| 43 | ASD | 0 | 0 | 1 | 0 |
| 44 | ASD | 0 | 0 | 2 | 0 |
| 45 | ASD | 1 | 0 | 2 | 0 |
| 46 | ASD | 1 | 0 | 2 | 0 |
| 47 | ASD | 0 | 0 | 2 | 0 |
| 48 | ASD | 0 | 0 | 2 | 0 |
| 49 | ASD | 1 | 0 | 2 | 0 |
| 50 | ASD | 1 | 1 | 1 | 0 |
| 51 | ASD | 1 | 0 | 2 | 0 |
| 52 | ASD | 1 | 0 | 2 | 0 |
| 53 | ASD | 1 | 0 | 2 | 0 |
| 54 | ASD | 1 | 1 | 1 | 0 |
| 55 | ASD | 1 | 1 | 1 | 0 |
| 56 | ASD | 1 | 0 | 1 | 1 |
| 57 | ASD | 0 | 0 | 2 | 0 |
| 58 | ASD | 1 | 0 | 2 | 0 |
| 59 | ASD | 0 | 0 | 2 | 0 |
| 60 | ASD | 0 | 1 | 2 | 0 |
| 61 | ASD | 1 | 1 | 2 | 0 |
| 62 | ASD | 1 | 0 | 1 | 0 |
| 63 | ASD | 0 | 0 | 2 | 0 |
| 64 | ASD | 1 | 1 | 1 | 0 |
| 65 | ASD | 1 | 0 | 2 | 0 |
| 66 | ASD | 0 | 0 | 4 | 0 |
| 67 | ASD | 0 | 1 | 1 | 0 |
| 68 | ASD | 1 | 1 | 2 | 0 |
| 69 | ASD | 1 | 1 | 1 | 0 |
| 70 | ASD | 0 | 0 | 1 | 0 |
| 71 | ASD | 1 | 0 | 2 | 0 |
| 72 | ASD | 0 | 0 | 2 | 0 |
| 73 | ASD | 1 | 0 | no response | 0 |

| | | | | | |
|---|---|---|---|---|---|
| 74 | ASD | 1 | 1 | 2 | 0 |
| 75 | ASD | 0 | 0 | 1 | 0 |
| 76 | ASD | 1 | 1 | 1 | 0 |
| 77 | ASD | 1 | 0 | 1 | 0 |
| 78 | ASD | 0 | 0 | 2 | 0 |
| 79 | ASD | 1 | 1 | 1 | 0 |
| 80 | ASD | 1 | 0 | 1 | 0 |
| 81 | ASD | 0 | 0 | 2 | 0 |
| 82 | ASD | 1 | 0 | 3 | 0 |
| 83 | ASD | 0 | 0 | 3 | 0 |
| 84 | ASD | 1 | 1 | 1 | 0 |
| 85 | ASD | 0 | 0 | 2 | 0 |
| 86 | ASD/GJH | 1 | 1 | 1 | 0 |
| 87 | ASD/GJH | 1 | 1 | 1 | 1 |
| 88 | ASD/GJH | 1 | 1 | 1 | 1 |
| 89 | ASD/GJH | 1 | 1 | 1 | 0 |
| 90 | ASD/GJH | 0 | 1 | 2 | 0 |
| 91 | ASD/GJH | 0 | 1 | 2 | 0 |
| 92 | ASD/GJH | 1 | 1 | 1 | 0 |
| 93 | ASD/GJH | 1 | 1 | 1 | 0 |
| 94 | ASD/GJH | 1 | 1 | 1 | 0 |
| 95 | ASD/GJH | 0 | 1 | 2 | 0 |
| 96 | ASD/GJH | 1 | 1 | 1 | 0 |
| 97 | ASD/GJH | 1 | 1 | 3 | 0 |
| 98 | ASD/GJH | 1 | 1 | 1 | 0 |
| 99 | ASD/GJH | 1 | 1 | 2 | 0 |
| 100 | ASD/GJH | 1 | 1 | 1 | 0 |
| 101 | ASD/GJH | 1 | 1 | 1 | 0 |

| | | | | | |
|---|---|---|---|---|---|
| 102 | ASD/GJH | 0 | 1 | 1 | 0 |
| 103 | ASD/GJH | 0 | 1 | 1 | 0 |
| 104 | ASD/GJH | 1 | 1 | 1 | 0 |
| 105 | ASD/GJH | 1 | 1 | 1 | 0 |
