# Supplementary material for: A Cohort Study Comparing Women with Autism Spectrum Disorder with and without Generalized Joint Hypermobility
Source: Behav Sci (Basel). 2018 Mar 17;8(3):35. doi: 10.3390/bs8030035 (PMC5867488; doi:10.3390/bs8030035)
Supplement: Supplementary file 1 [file behavsci-08-00035-s001.zip › Supplementary_Files/S2.docx]

**SUPPLEMENTARY TABLES**

**Supplementary Table 1.** Immune symptoms: hospitalization history, asthma, ear infections, rhinitis, and sinusitis. 0 = no symptom reported; 1 = symptom reported.

| **ID#** | **Group** | **Hospitalization** | **Asthma** | **Ear Infections** | **Rhinitis** | **Sinusitis** |
| --- | --- | --- | --- | --- | --- | --- |
| 1 | ASD | 0 | 0 | 0 | 0 | 0 |
| 2 | ASD | 0 | 0 | 0 | 0 | 1 |
| 3 | ASD | 0 | 0 | 0 | 0 | 0 |
| 4 | ASD | 0 | 0 | 0 | 0 | 0 |
| 5 | ASD | 1 | 1 | 0 | 0 | 0 |
| 6 | ASD | 1 | 1 | 0 | 0 | 0 |
| 7 | ASD | 1 | 0 | 0 | 0 | 0 |
| 8 | ASD | 0 | 0 | 0 | 1 | 1 |
| 9 | ASD | 0 | 1 | 0 | 1 | 0 |
| 10 | ASD | 0 | 0 | 0 | 0 | 1 |
| 11 | ASD | 0 | 0 | 0 | 0 | 0 |
| 12 | ASD | 0 | 1 | 0 | 0 | 0 |
| 13 | ASD | 1 | 1 | 0 | 1 | 0 |
| 14 | ASD | 1 | 0 | 0 | 0 | 0 |
| 15 | ASD | 0 | 1 | 0 | 0 | 0 |
| 16 | ASD | 1 | 1 | 1 | 1 | 1 |
| 17 | ASD | 1 | 0 | 0 | 0 | 0 |
| 18 | ASD | 1 | 1 | 0 | 1 | 0 |
| 19 | ASD | 0 | 0 | 1 | 1 | 1 |
| 20 | ASD | 0 | 0 | 0 | 0 | 1 |
| 21 | ASD | 0 | 1 | 0 | 0 | 0 |
| 22 | ASD | 1 | 0 | 0 | 1 | 0 |
| 23 | ASD | 1 | 1 | 1 | 0 | 1 |
| 24 | ASD | 0 | 0 | 0 | 0 | 0 |
| 25 | ASD | 0 | 1 | 0 | 0 | 0 |
| 26 | ASD | 1 | 1 | 1 | 0 | 0 |
| 27 | ASD | 0 | 1 | 0 | 1 | 1 |
| 28 | ASD | 0 | 1 | 1 | 1 | 1 |
| 29 | ASD | 0 | 1 | 1 | 1 | 1 |
| 30 | ASD | 0 | 0 | 0 | 0 | 0 |
| 31 | ASD | 0 | 0 | 1 | 0 | 0 |
| 32 | ASD | 0 | 0 | 1 | 1 | 1 |
| 33 | ASD | 0 | 0 | 0 | 0 | 0 |
| 34 | ASD | 0 | 0 | 1 | 0 | 0 |
| 35 | ASD | 0 | 0 | 1 | 1 | 1 |
| 36 | ASD | 0 | 0 | 0 | 0 | 0 |
| 37 | ASD | 0 | 0 | 1 | 1 | 1 |
| 38 | ASD | 1 | 0 | 1 | 0 | 1 |
| 39 | ASD | 0 | 0 | 0 | 0 | 0 |
| 40 | ASD | 0 | 0 | 1 | 1 | 1 |
| 41 | ASD | 1 | 1 | 1 | 1 | 0 |
| 42 | ASD | 0 | 0 | 1 | 0 | 0 |
| 43 | ASD | 1 | 1 | 0 | 0 | 0 |
| 44 | ASD | 0 | 0 | 0 | 0 | 0 |
| 45 | ASD | 1 | 0 | 0 | 0 | 1 |
| 46 | ASD | 0 | 0 | 0 | 0 | 0 |
| 47 | ASD | 0 | 0 | 1 | 0 | 1 |
| 48 | ASD | 0 | 0 | 0 | 0 | 0 |
| 49 | ASD | 0 | 0 | 0 | 0 | 0 |
| 50 | ASD | 0 | 0 | 0 | 0 | 0 |
| 51 | ASD | 0 | 0 | 1 | 1 | 1 |
| 52 | ASD | 0 | 0 | 1 | 0 | 0 |
| 53 | ASD | 0 | 1 | 0 | 0 | 0 |
| 54 | ASD | 0 | 0 | 0 | 1 | 1 |
| 55 | ASD | 1 | 1 | 1 | 1 | 1 |
| 56 | ASD | 1 | 1 | 0 | 1 | 1 |
| 57 | ASD | 0 | 1 | 0 | 0 | 0 |
| 58 | ASD | 0 | 0 | 0 | 0 | 0 |
| 59 | ASD | 0 | 0 | 0 | 0 | 0 |
| 60 | ASD | 0 | 0 | 1 | 1 | 1 |
| 61 | ASD | 0 | 1 | 1 | 0 | 1 |
| 62 | ASD | 0 | 1 | 1 | 1 | 1 |
| 63 | ASD | 0 | 0 | 1 | 1 | 1 |
| 64 | ASD | 0 | 0 | 0 | 0 | 1 |
| 65 | ASD | 0 | 0 | 0 | 0 | 1 |
| 66 | ASD | 0 | 0 | 0 | 1 | 0 |
| 67 | ASD | 1 | 1 | 1 | 1 | 1 |
| 68 | ASD | 0 | 1 | 1 | 0 | 1 |
| 69 | ASD | 0 | 0 | 1 | 1 | 1 |
| 70 | ASD | 0 | 0 | 0 | 0 | 0 |
| 71 | ASD | 0 | 0 | 0 | 1 | 1 |
| 72 | ASD | 1 | 0 | 1 | 0 | 0 |
| 73 | ASD | 0 | 1 | 1 | 1 | 1 |
| 74 | ASD | 1 | 0 | 0 | 0 | 0 |
| 75 | ASD | 1 | 0 | 0 | 1 | 1 |
| 76 | ASD | 1 | 0 | 0 | 0 | 1 |
| 77 | ASD | 0 | 0 | 0 | 1 | 1 |
| 78 | ASD | 0 | 0 | 1 | 0 | 0 |
| 79 | ASD | 1 | 0 | 1 | 1 | 1 |
| 80 | ASD | 0 | 1 | 1 | 1 | 1 |
| 81 | ASD | 1 | 0 | 1 | 1 | 1 |
| 82 | ASD | 0 | 0 | 1 | 0 | 0 |
| 83 | ASD | 0 | 1 | 1 | 0 | 1 |
| 84 | ASD | 0 | 0 | 0 | 0 | 0 |
| 85 | ASD | 0 | 0 | 0 | 0 | 0 |
| 86 | ASD/GJH | 0 | 1 | 1 | 1 | 0 |
| 87 | ASD/GJH | 1 | 1 | 1 | 1 | 1 |
| 88 | ASD/GJH | 0 | 1 | 0 | 1 | 1 |
| 89 | ASD/GJH | 0 | 0 | 1 | 1 | 1 |
| 90 | ASD/GJH | 1 | 1 | 0 | 0 | 0 |
| 91 | ASD/GJH | 0 | 1 | 1 | 1 | 1 |
| 92 | ASD/EDS | 1 | 1 | 1 | 1 | 1 |
| 93 | ASD/GJH | 1 | 1 | 1 | 1 | 0 |
| 94 | ASD/GJH | 0 | 1 | 1 | 1 | 1 |
| 95 | ASD/GJH | 1 | 1 | 0 | 0 | 1 |
| 96 | ASD/GJH | 0 | 0 | 0 | 0 | 0 |
| 97 | ASD/GJH | 1 | 0 | 0 | 0 | 0 |
| 98 | ASD/GJH | 1 | 1 | 1 | 1 | 1 |
| 99 | ASD/GJH | 0 | 0 | 1 | 0 | 1 |
| 100 | ASD/GJH | 0 | 0 | 0 | 1 | 1 |
| 101 | ASD/GJH | 1 | 1 | 1 | 1 | 1 |
| 102 | ASD/GJH | 1 | 0 | 1 | 0 | 0 |
| 103 | ASD/GJH | 0 | 1 | 0 | 0 | 0 |
| 104 | ASD/GJH | 1 | 0 | 1 | 0 | 0 |
| 105 | ASD/GJH | 0 | 0 | 1 | 1 | 1 |

**Supplementary Table 2.** Immune symptoms: allergies, reaction to medications (med react), reaction to environmental chemicals (env react), autoimmunity, and sum of all immune symptoms. 0 = no symptom reported; 1 = symptom reported.

| **ID#** | **Group** | **Allergies** | **Med React** | **Env React** | **Autoimmune** | **All**  **(Sum of) Immune** |
| --- | --- | --- | --- | --- | --- | --- |
| 1 | ASD | 0 | 0 | 0 | 0 | 0 |
| 2 | ASD | 0 | 0 | 0 | 0 | 1 |
| 3 | ASD | 0 | 0 | 0 | 0 | 0 |
| 4 | ASD | 1 | 0 | 0 | 0 | 1 |
| 5 | ASD | 0 | 0 | 0 | 0 | 2 |
| 6 | ASD | 0 | 0 | 0 | 0 | 2 |
| 7 | ASD | 0 | 0 | 0 | 0 | 1 |
| 8 | ASD | 0 | 1 | 1 | 0 | 4 |
| 9 | ASD | 0 | 0 | 1 | 0 | 3 |
| 10 | ASD | 0 | 0 | 0 | 0 | 1 |
| 11 | ASD | 0 | 0 | 0 | 0 | 0 |
| 12 | ASD | 0 | 0 | 0 | 0 | 1 |
| 13 | ASD | 1 | 1 | 1 | 0 | 6 |
| 14 | ASD | 0 | 0 | 0 | 0 | 1 |
| 15 | ASD | 1 | 0 | 1 | 0 | 3 |
| 16 | ASD | 1 | 1 | 1 | 1 | 9 |
| 17 | ASD | 0 | 0 | 0 | 0 | 1 |
| 18 | ASD | 0 | 0 | 0 | 0 | 3 |
| 19 | ASD | 0 | 0 | 1 | 0 | 4 |
| 20 | ASD | 0 | 1 | 1 | 0 | 3 |
| 21 | ASD | 0 | 0 | 0 | 1 | 2 |
| 22 | ASD | 1 | 0 | 1 | 0 | 4 |
| 23 | ASD | 1 | 1 | 1 | 0 | 7 |
| 24 | ASD | 0 | 0 | 0 | 0 | 0 |
| 25 | ASD | 0 | 0 | 0 | 0 | 1 |
| 26 | ASD | 1 | 0 | 0 | 1 | 5 |
| 27 | ASD | 1 | 1 | 1 | 0 | 6 |
| 28 | ASD | 1 | 0 | 0 | 1 | 6 |
| 29 | ASD | 1 | 1 | 1 | 0 | 7 |
| 30 | ASD | 0 | 1 | 1 | 0 | 2 |
| 31 | ASD | 0 | 0 | 0 | 0 | 1 |
| 32 | ASD | 0 | 0 | 0 | 0 | 3 |
| 33 | ASD | 0 | 0 | 0 | 0 | 0 |
| 34 | ASD | 0 | 0 | 0 | 1 | 2 |
| 35 | ASD | 1 | 1 | 1 | 0 | 6 |
| 36 | ASD | 0 | 1 | 0 | 0 | 1 |
| 37 | ASD | 0 | 0 | 1 | 0 | 4 |
| 38 | ASD | 1 | 1 | 1 | 0 | 6 |
| 39 | ASD | 0 | 0 | 1 | 0 | 1 |
| 40 | ASD | 1 | 0 | 0 | 0 | 4 |
| 41 | ASD | 1 | 1 | 1 | 0 | 7 |
| 42 | ASD | 1 | 1 | 0 | 0 | 3 |
| 43 | ASD | 1 | 0 | 1 | 0 | 4 |
| 44 | ASD | 0 | 0 | 0 | 0 | 0 |
| 45 | ASD | 0 | 1 | 0 | 0 | 3 |
| 46 | ASD | 0 | 0 | 0 | 0 | 0 |
| 47 | ASD | 0 | 0 | 0 | 0 | 2 |
| 48 | ASD | 0 | 0 | 0 | 0 | 0 |
| 49 | ASD | 0 | 0 | 0 | 0 | 0 |
| 50 | ASD | 1 | 0 | 0 | 0 | 1 |
| 51 | ASD | 0 | 0 | 0 | 0 | 3 |
| 52 | ASD | 0 | 0 | 0 | 0 | 1 |
| 53 | ASD | 0 | 0 | 1 | 0 | 2 |
| 54 | ASD | 1 | 0 | 0 | 0 | 3 |
| 55 | ASD | 1 | 1 | 1 | 0 | 8 |
| 56 | ASD | 1 | 1 | 1 | 0 | 7 |
| 57 | ASD | 0 | 0 | 0 | 1 | 2 |
| 58 | ASD | 0 | 1 | 0 | 0 | 1 |
| 59 | ASD | 1 | 0 | 0 | 0 | 1 |
| 60 | ASD | 1 | 0 | 1 | 0 | 5 |
| 61 | ASD | 0 | 1 | 1 | 0 | 5 |
| 62 | ASD | 1 | 1 | 1 | 0 | 7 |
| 63 | ASD | 0 | 1 | 0 | 0 | 4 |
| 64 | ASD | 1 | 1 | 1 | 0 | 4 |
| 65 | ASD | 0 | 0 | 0 | 0 | 1 |
| 66 | ASD | 1 | 0 | 0 | 0 | 2 |
| 67 | ASD | 1 | 0 | 0 | 1 | 7 |
| 68 | ASD | 1 | 1 | 0 | 0 | 5 |
| 69 | ASD | 1 | 1 | 1 | 0 | 6 |
| 70 | ASD | 0 | 0 | 0 | 1 | 1 |
| 71 | ASD | 1 | 0 | 1 | 0 | 4 |
| 72 | ASD | 1 | 0 | 0 | 0 | 3 |
| 73 | ASD | 1 | 1 | 0 | 1 | 7 |
| 74 | ASD | 1 | 0 | 1 | 1 | 4 |
| 75 | ASD | 1 | 1 | 1 | 0 | 6 |
| 76 | ASD | 1 | 1 | 1 | 0 | 5 |
| 77 | ASD | 0 | 0 | 0 | 0 | 2 |
| 78 | ASD | 0 | 0 | 0 | 0 | 1 |
| 79 | ASD | 1 | 1 | 1 | 1 | 8 |
| 80 | ASD | 1 | 1 | 1 | 0 | 7 |
| 81 | ASD | 1 | 1 | 0 | 0 | 6 |
| 82 | ASD | 0 | 0 | 0 | 0 | 1 |
| 83 | ASD | 0 | 0 | 1 | 0 | 4 |
| 84 | ASD | 1 | 0 | 0 | 0 | 1 |
| 85 | ASD | 0 | 1 | 0 | 0 | 1 |
| 86 | ASD/GJH | 0 | 1 | 1 | 0 | 5 |
| 87 | ASD/GJH | 1 | 1 | 1 | 1 | 9 |
| 88 | ASD/GJH | 1 | 0 | 1 | 0 | 5 |
| 89 | ASD/GJH | 0 | 1 | 0 | 1 | 5 |
| 90 | ASD/GJH | 1 | 0 | 1 | 0 | 4 |
| 91 | ASD/GJH | 1 | 0 | 0 | 0 | 5 |
| 92 | ASD/GJH | 1 | 1 | 1 | 0 | 8 |
| 93 | ASD/GJH | 0 | 0 | 0 | 1 | 5 |
| 94 | ASD/GJH | 1 | 1 | 1 | 1 | 8 |
| 95 | ASD/GJH | 0 | 1 | 1 | 0 | 5 |
| 96 | ASD/GJH | 1 | 1 | 0 | 1 | 3 |
| 97 | ASD/GJH | 0 | 1 | 1 | 0 | 3 |
| 98 | ASD/GJH | 1 | 1 | 1 | 1 | 9 |
| 99 | ASD/GJH | 1 | 1 | 1 | 0 | 5 |
| 100 | ASD/GJH | 1 | 1 | 1 | 0 | 5 |
| 101 | ASD/GJH | 1 | 1 | 1 | 1 | 9 |
| 102 | ASD/GJH | 0 | 0 | 0 | 1 | 3 |
| 103 | ASD/GJH | 1 | 0 | 0 | 0 | 2 |
| 104 | ASD/GJH | 0 | 1 | 1 | 1 | 5 |
| 105 | ASD/GJH | 0 | 0 | 0 | 0 | 3 |

**Supplementary Table 3.** Endocrine symptoms: polycystic ovary syndrome (PCOS), amenorrhea, diabetes 2/insulin resistance (diabetes, endometriosis, and adult acne. 0 = no symptom reported; 1 = symptom reported.

| **ID#** | **Group** | **PCOS** | **Amenorrhea** | **Diabetes** | **Endometriosis** | **Adult Acne** |
| --- | --- | --- | --- | --- | --- | --- |
| 1 | ASD | 0 | 0 | 0 | 0 | 0 |
| 2 | ASD | 0 | 0 | 0 | 0 | 0 |
| 3 | ASD | 0 | 0 | 0 | 0 | 0 |
| 4 | ASD | 0 | 0 | 0 | 0 | 0 |
| 5 | ASD | 0 | 0 | 0 | 0 | 0 |
| 6 | ASD | 0 | 1 | 0 | 0 | 0 |
| 7 | ASD | 0 | 0 | 0 | 0 | 1 |
| 8 | ASD | 0 | 0 | 0 | 0 | 0 |
| 9 | ASD | 0 | 0 | 1 | 0 | 0 |
| 10 | ASD | 0 | 0 | 0 | 0 | 0 |
| 11 | ASD | 0 | 0 | 0 | 0 | 0 |
| 12 | ASD | 0 | 1 | 0 | 0 | 0 |
| 13 | ASD | 0 | 0 | 0 | 0 | 0 |
| 14 | ASD | 0 | 1 | 0 | 0 | 0 |
| 15 | ASD | 0 | 1 | 0 | 0 | 1 |
| 16 | ASD | 0 | 1 | 0 | 0 | 1 |
| 17 | ASD | 0 | 0 | 0 | 0 | 0 |
| 18 | ASD | 0 | 1 | 0 | 0 | 1 |
| 19 | ASD | 0 | 0 | 1 | 0 | 0 |
| 20 | ASD | 0 | 1 | 0 | 0 | 0 |
| 21 | ASD | 0 | 0 | 0 | 0 | 0 |
| 22 | ASD | 0 | 1 | 0 | 0 | 0 |
| 23 | ASD | 0 | 0 | 0 | 0 | 0 |
| 24 | ASD | 0 | 1 | 0 | 0 | 0 |
| 25 | ASD | 0 | 0 | 0 | 0 | 1 |
| 26 | ASD | 0 | 1 | 0 | 0 | 1 |
| 27 | ASD | 0 | 0 | 0 | 0 | 0 |
| 28 | ASD | 1 | 1 | 1 | 0 | 0 |
| 29 | ASD | 0 | 1 | 0 | 0 | 0 |
| 30 | ASD | 0 | 1 | 0 | 0 | 1 |
| 31 | ASD | 0 | 1 | 0 | 0 | 0 |
| 32 | ASD | 0 | 0 | 0 | 0 | 0 |
| 33 | ASD | 0 | 0 | 0 | 0 | 1 |
| 34 | ASD | 0 | 0 | 0 | 0 | 0 |
| 35 | ASD | 1 | 0 | 0 | 1 | 1 |
| 36 | ASD | 0 | 0 | 0 | 0 | 0 |
| 37 | ASD | 0 | 0 | 0 | 0 | 0 |
| 38 | ASD | 0 | 0 | 0 | 1 | 0 |
| 39 | ASD | 0 | 1 | 0 | 0 | 0 |
| 40 | ASD | 0 | 0 | 0 | 0 | 0 |
| 41 | ASD | 0 | 1 | 0 | 0 | 1 |
| 42 | ASD | 0 | 0 | 0 | 0 | 1 |
| 43 | ASD | 0 | 1 | 0 | 0 | 0 |
| 44 | ASD | 0 | 0 | 0 | 0 | 0 |
| 45 | ASD | 0 | 0 | 0 | 0 | 0 |
| 46 | ASD | 0 | 0 | 0 | 0 | 0 |
| 47 | ASD | 0 | 0 | 0 | 0 | 0 |
| 48 | ASD | 0 | 0 | 0 | 0 | 0 |
| 49 | ASD | 0 | 0 | 0 | 0 | 0 |
| 50 | ASD | 0 | 0 | 0 | 0 | 0 |
| 51 | ASD | 0 | 0 | 0 | 0 | 0 |
| 52 | ASD | 0 | 0 | 0 | 0 | 0 |
| 53 | ASD | 0 | 0 | 0 | 0 | 0 |
| 54 | ASD | 0 | 0 | 0 | 0 | 1 |
| 55 | ASD | 1 | 1 | 1 | 0 | 0 |
| 56 | ASD | 0 | 0 | 0 | 0 | 0 |
| 57 | ASD | 0 | 0 | 0 | 0 | 0 |
| 58 | ASD | 1 | 1 | 1 | 0 | 1 |
| 59 | ASD | 0 | 1 | 0 | 0 | 0 |
| 60 | ASD | 0 | 1 | 0 | 1 | 1 |
| 61 | ASD | 0 | 1 | 0 | 0 | 0 |
| 62 | ASD | 0 | 0 | 0 | 0 | 0 |
| 63 | ASD | 0 | 0 | 0 | 0 | 0 |
| 64 | ASD | 0 | 1 | 0 | 0 | 0 |
| 65 | ASD | 0 | 1 | 0 | 0 | 0 |
| 66 | ASD | 0 | 0 | 0 | 0 | 0 |
| 67 | ASD | 0 | 1 | 0 | 0 | 0 |
| 68 | ASD | 1 | 1 | 0 | 0 | 1 |
| 69 | ASD | 1 | 0 | 0 | 0 | 0 |
| 70 | ASD | 0 | 0 | 0 | 0 | 0 |
| 71 | ASD | 0 | 0 | 0 | 0 | 0 |
| 72 | ASD | 0 | 1 | 0 | 0 | 0 |
| 73 | ASD | 0 | 0 | 0 | 0 | 0 |
| 74 | ASD | 0 | 0 | 0 | 0 | 1 |
| 75 | ASD | 0 | 1 | 0 | 0 | 0 |
| 76 | ASD | 0 | 1 | 0 | 1 | 0 |
| 77 | ASD | 0 | 0 | 0 | 0 | 1 |
| 78 | ASD | 0 | 1 | 0 | 0 | 0 |
| 79 | ASD | 0 | 1 | 0 | 0 | 0 |
| 80 | ASD | 0 | 0 | 0 | 0 | 0 |
| 81 | ASD | 0 | 0 | 0 | 0 | 0 |
| 82 | ASD | 0 | 0 | 0 | 0 | 0 |
| 83 | ASD | 0 | 1 | 0 | 0 | 0 |
| 84 | ASD | 1 | 1 | 0 | 0 | 1 |
| 85 | ASD | 0 | 0 | 0 | 0 | 0 |
| 86 | ASD/GJH | 0 | 1 | 0 | 0 | 1 |
| 87 | ASD/GJH | 0 | 1 | 0 | 0 | 0 |
| 88 | ASD/GJH | 0 | 1 | 0 | 0 | 0 |
| 89 | ASD/GJH | 0 | 0 | 0 | 1 | 1 |
| 90 | ASD/GJH | 0 | 0 | 0 | 0 | 0 |
| 91 | ASD/GJH | 0 | 0 | 0 | 0 | 0 |
| 92 | ASD/GJH | 0 | 0 | 0 | 0 | 1 |
| 93 | ASD/GJH | 1 | 1 | 0 | 0 | 0 |
| 94 | ASD/GJH | 0 | 1 | 1 | 1 | 1 |
| 95 | ASD/GJH | 0 | 0 | 0 | 0 | 0 |
| 96 | ASD/GJH | 0 | 1 | 0 | 0 | 0 |
| 97 | ASD/GJH | 0 | 1 | 0 | 1 | 0 |
| 98 | ASD/GJH | 1 | 1 | 0 | 1 | 1 |
| 99 | ASD/GJH | 0 | 0 | 0 | 0 | 0 |
| 100 | ASD/GJH | 0 | 0 | 0 | 0 | 0 |
| 101 | ASD/GJH | 1 | 1 | 0 | 1 | 1 |
| 102 | ASD/GJH | 0 | 0 | 0 | 0 | 0 |
| 103 | ASD/GJH | 0 | 0 | 0 | 1 | 0 |
| 104 | ASD/GJH | 1 | 0 | 0 | 0 | 1 |
| 105 | ASD/GJH | 1 | 0 | 1 | 0 | 0 |

**Supplementary File 4.** Endocrine symptoms: infertility, dysmenorrhea, irregular menses, high LDL cholesterol, and hypertension. 0 = no symptom reported; 1 = symptom reported.

| **ID#** | **Group** | **Infertility** | **Dysmeno-rrhea** | **Irreg. Menses** | **High LDL** | **Hypertension** |
| --- | --- | --- | --- | --- | --- | --- |
| 1 | ASD | 0 | 0 | 0 | 0 | 0 |
| 2 | ASD | 0 | 0 | 0 | 0 | 1 |
| 3 | ASD | 1 | 0 | 0 | 0 | 0 |
| 4 | ASD | 0 | 0 | 0 | 0 | 0 |
| 5 | ASD | 0 | 0 | 0 | 0 | 0 |
| 6 | ASD | 1 | 1 | 0 | 0 | 0 |
| 7 | ASD | 0 | 1 | 0 | 0 | 0 |
| 8 | ASD | 0 | 0 | 0 | 0 | 0 |
| 9 | ASD | 0 | 0 | 0 | 1 | 0 |
| 10 | ASD | 0 | 0 | 0 | 0 | 1 |
| 11 | ASD | 0 | 0 | 0 | 0 | 0 |
| 12 | ASD | 0 | 0 | 0 | 0 | 0 |
| 13 | ASD | 0 | 0 | 0 | 0 | 0 |
| 14 | ASD | 0 | 0 | 0 | 0 | 0 |
| 15 | ASD | 0 | 0 | 0 | 0 | 0 |
| 16 | ASD | 0 | 0 | 1 | 1 | 0 |
| 17 | ASD | 0 | 0 | 0 | 0 | 0 |
| 18 | ASD | 0 | 1 | 1 | 1 | 1 |
| 19 | ASD | 0 | 0 | 0 | 0 | 0 |
| 20 | ASD | 0 | 1 | 1 | 0 | 0 |
| 21 | ASD | 0 | 0 | 0 | 0 | 0 |
| 22 | ASD | 0 | 0 | 0 | 0 | 0 |
| 23 | ASD | 0 | 1 | 0 | 0 | 0 |
| 24 | ASD | 0 | 0 | 0 | 0 | 0 |
| 25 | ASD | 0 | 1 | 0 | 0 | 0 |
| 26 | ASD | 0 | 0 | 1 | 0 | 0 |
| 27 | ASD | 0 | 1 | 0 | 1 | 0 |
| 28 | ASD | 0 | 0 | 1 | 1 | 1 |
| 29 | ASD | 0 | 1 | 1 | 1 | 0 |
| 30 | ASD | 0 | 0 | 1 | 0 | 0 |
| 31 | ASD | 0 | 0 | 1 | 0 | 0 |
| 32 | ASD | 0 | 0 | 0 | 0 | 0 |
| 33 | ASD | 0 | 0 | 0 | 0 | 0 |
| 34 | ASD | 0 | 1 | 0 | 0 | 0 |
| 35 | ASD | 1 | 0 | 0 | 0 | 0 |
| 36 | ASD | 0 | 1 | 0 | 0 | 1 |
| 37 | ASD | 0 | 0 | 0 | 0 | 0 |
| 38 | ASD | 0 | 1 | 0 | 0 | 0 |
| 39 | ASD | 0 | 0 | 0 | 1 | 1 |
| 40 | ASD | 0 | 0 | 0 | 0 | 0 |
| 41 | ASD | 0 | 1 | 1 | 0 | 0 |
| 42 | ASD | 0 | 1 | 0 | 0 | 0 |
| 43 | ASD | 0 | 1 | 0 | 0 | 0 |
| 44 | ASD | 0 | 1 | 0 | 0 | 0 |
| 45 | ASD | 0 | 0 | 0 | 1 | 0 |
| 46 | ASD | 0 | 0 | 0 | 0 | 0 |
| 47 | ASD | 0 | 0 | 0 | 0 | 0 |
| 48 | ASD | 0 | 0 | 0 | 0 | 0 |
| 49 | ASD | 0 | 0 | 0 | 0 | 0 |
| 50 | ASD | 0 | 1 | 1 | 0 | 0 |
| 51 | ASD | 0 | 0 | 0 | 0 | 0 |
| 52 | ASD | 0 | 0 | 0 | 0 | 0 |
| 53 | ASD | 0 | 0 | 0 | 0 | 0 |
| 54 | ASD | 0 | 1 | 0 | 0 | 0 |
| 55 | ASD | 1 | 0 | 1 | 1 | 1 |
| 56 | ASD | 0 | 0 | 1 | 0 | 0 |
| 57 | ASD | 0 | 0 | 0 | 0 | 1 |
| 58 | ASD | 0 | 0 | 1 | 0 | 0 |
| 59 | ASD | 0 | 0 | 0 | 0 | 0 |
| 60 | ASD | 0 | 1 | 1 | 0 | 0 |
| 61 | ASD | 0 | 0 | 0 | 0 | 0 |
| 62 | ASD | 0 | 0 | 0 | 0 | 0 |
| 63 | ASD | 0 | 1 | 1 | 0 | 0 |
| 64 | ASD | 0 | 0 | 0 | 0 | 0 |
| 65 | ASD | 0 | 1 | 1 | 0 | 0 |
| 66 | ASD | 1 | 0 | 0 | 0 | 0 |
| 67 | ASD | 0 | 0 | 1 | 0 | 0 |
| 68 | ASD | 0 | 0 | 1 | 1 | 0 |
| 69 | ASD | 1 | 0 | 0 | 0 | 0 |
| 70 | ASD | 0 | 0 | 0 | 0 | 0 |
| 71 | ASD | 0 | 0 | 0 | 0 | 0 |
| 72 | ASD | 0 | 0 | 0 | 0 | 0 |
| 73 | ASD | 0 | 0 | 0 | 1 | 1 |
| 74 | ASD | 0 | 1 | 0 | 0 | 0 |
| 75 | ASD | 0 | 0 | 0 | 0 | 1 |
| 76 | ASD | 0 | 1 | 1 | 0 | 1 |
| 77 | ASD | 0 | 0 | 0 | 1 | 0 |
| 78 | ASD | 0 | 0 | 1 | 0 | 0 |
| 79 | ASD | 0 | 0 | 1 | 0 | 0 |
| 80 | ASD | 0 | 0 | 0 | 0 | 1 |
| 81 | ASD | 0 | 0 | 0 | 0 | 0 |
| 82 | ASD | 0 | 0 | 0 | 0 | 0 |
| 83 | ASD | 0 | 0 | 1 | 0 | 0 |
| 84 | ASD | 1 | 1 | 1 | 0 | 0 |
| 85 | ASD | 0 | 1 | 0 | 0 | 0 |
| 86 | ASD/GJH | 1 | 1 | 1 | 0 | 0 |
| 87 | ASD/GJH | 0 | 1 | 0 | 0 | 0 |
| 88 | ASD/GJH | 0 | 0 | 1 | 0 | 0 |
| 89 | ASD/GJH | 0 | 1 | 0 | 0 | 0 |
| 90 | ASD/GJH | 0 | 0 | 0 | 0 | 0 |
| 91 | ASD/GJH | 0 | 1 | 0 | 1 | 1 |
| 92 | ASD/GJH | 0 | 1 | 0 | 0 | 0 |
| 93 | ASD/GJH | 0 | 1 | 1 | 0 | 1 |
| 94 | ASD/GJH | 0 | 1 | 1 | 1 | 1 |
| 95 | ASD/GJH | 0 | 1 | 0 | 1 | 1 |
| 96 | ASD/GJH | 0 | 1 | 1 | 0 | 0 |
| 97 | ASD/GJH | 0 | 1 | 1 | 1 | 0 |
| 98 | ASD/GJH | 0 | 1 | 1 | 0 | 0 |
| 99 | ASD/GJH | 1 | 0 | 0 | 0 | 0 |
| 100 | ASD/GJH | 0 | 1 | 1 | 1 | 0 |
| 101 | ASD/GJH | 0 | 1 | 1 | 0 | 0 |
| 102 | ASD/GJH | 0 | 1 | 0 | 0 | 0 |
| 103 | ASD/GJH | 1 | 1 | 0 | 0 | 0 |
| 104 | ASD/GJH | 0 | 1 | 1 | 0 | 0 |
| 105 | ASD/GJH | 0 | 1 | 1 | 1 | 0 |

**Supplementary Table 5.** Endocrine symptoms: hirsutism, overweight/obesity, premenstrual dysphoric disorder (PMDD), severe teen acne, uterine fibroids, and sum of all endocrine symptoms. 0 = no symptom reported; 1 = symptom reported.

| **ID#** | **Group** | **Hirsutism** | **Overweight/**  **Obesity** | **PMDD** | **Severe Teen Acne** | **Uterine**  **Fibroids** | **All**  **(Sum of ) Endo** |
| --- | --- | --- | --- | --- | --- | --- | --- |
| 1 | ASD | 0 | 0 | 1 | 0 | 0 | 1 |
| 2 | ASD | 0 | 0 | 0 | 0 | 0 | 1 |
| 3 | ASD | 0 | 0 | 0 | 0 | 0 | 1 |
| 4 | ASD | 0 | 0 | 0 | 0 | 0 | 0 |
| 5 | ASD | 1 | 0 | 0 | 0 | 0 | 1 |
| 6 | ASD | 0 | 0 | 0 | 0 | 1 | 4 |
| 7 | ASD | 0 | 1 | 1 | 1 | 1 | 6 |
| 8 | ASD | 0 | 0 | 0 | 0 | 0 | 0 |
| 9 | ASD | 1 | 1 | 0 | 0 | 0 | 4 |
| 10 | ASD | 0 | 0 | 0 | 0 | 0 | 1 |
| 11 | ASD | 0 | 0 | 0 | 0 | 0 | 0 |
| 12 | ASD | 0 | 0 | 0 | 0 | 0 | 1 |
| 13 | ASD | 0 | 0 | 0 | 0 | 0 | 0 |
| 14 | ASD | 0 | 0 | 1 | 0 | 0 | 2 |
| 15 | ASD | 0 | 1 | 0 | 0 | 0 | 3 |
| 16 | ASD | 0 | 1 | 1 | 1 | 0 | 7 |
| 17 | ASD | 0 | 0 | 0 | 0 | 0 | 0 |
| 18 | ASD | 0 | 1 | 0 | 0 | 0 | 7 |
| 19 | ASD | 0 | 0 | 0 | 0 | 0 | 1 |
| 20 | ASD | 0 | 0 | 0 | 0 | 0 | 3 |
| 21 | ASD | 0 | 1 | 0 | 0 | 0 | 1 |
| 22 | ASD | 0 | 0 | 0 | 0 | 0 | 1 |
| 23 | ASD | 0 | 0 | 1 | 0 | 0 | 2 |
| 24 | ASD | 0 | 0 | 0 | 0 | 0 | 1 |
| 25 | ASD | 0 | 1 | 0 | 0 | 0 | 3 |
| 26 | ASD | 1 | 0 | 1 | 1 | 0 | 6 |
| 27 | ASD | 0 | 1 | 0 | 0 | 0 | 3 |
| 28 | ASD | 1 | 1 | 0 | 0 | 0 | 8 |
| 29 | ASD | 0 | 0 | 0 | 0 | 0 | 4 |
| 30 | ASD | 0 | 0 | 1 | 1 | 0 | 5 |
| 31 | ASD | 1 | 1 | 0 | 0 | 0 | 4 |
| 32 | ASD | 0 | 0 | 0 | 0 | 0 | 0 |
| 33 | ASD | 0 | 0 | 1 | 1 | 0 | 3 |
| 34 | ASD | 0 | 0 | 0 | 0 | 1 | 2 |
| 35 | ASD | 0 | 0 | 0 | 0 | 0 | 4 |
| 36 | ASD | 0 | 1 | 0 | 0 | 0 | 3 |
| 37 | ASD | 1 | 1 | 0 | 0 | 0 | 2 |
| 38 | ASD | 0 | 0 | 0 | 0 | 1 | 3 |
| 39 | ASD | 0 | 0 | 0 | 0 | 0 | 3 |
| 40 | ASD | 0 | 0 | 0 | 0 | 0 | 0 |
| 41 | ASD | 0 | 0 | 1 | 0 | 0 | 5 |
| 42 | ASD | 1 | 0 | 0 | 0 | 0 | 3 |
| 43 | ASD | 0 | 1 | 0 | 0 | 0 | 3 |
| 44 | ASD | 0 | 1 | 0 | 0 | 0 | 2 |
| 45 | ASD | 0 | 1 | 0 | 0 | 0 | 2 |
| 46 | ASD | 0 | 1 | 1 | 0 | 0 | 2 |
| 47 | ASD | 0 | 0 | 0 | 0 | 0 | 0 |
| 48 | ASD | 0 | 0 | 0 | 0 | 0 | 0 |
| 49 | ASD | 0 | 0 | 0 | 0 | 0 | 0 |
| 50 | ASD | 1 | 0 | 0 | 1 | 0 | 4 |
| 51 | ASD | 0 | 0 | 0 | 0 | 0 | 0 |
| 52 | ASD | 1 | 0 | 0 | 1 | 0 | 2 |
| 53 | ASD | 0 | 0 | 0 | 0 | 0 | 0 |
| 54 | ASD | 0 | 0 | 0 | 0 | 0 | 2 |
| 55 | ASD | 0 | 1 | 1 | 0 | 0 | 9 |
| 56 | ASD | 0 | 0 | 1 | 0 | 0 | 2 |
| 57 | ASD | 0 | 1 | 0 | 0 | 0 | 2 |
| 58 | ASD | 1 | 0 | 0 | 1 | 0 | 7 |
| 59 | ASD | 0 | 0 | 0 | 0 | 0 | 1 |
| 60 | ASD | 0 | 0 | 0 | 1 | 0 | 6 |
| 61 | ASD | 0 | 0 | 0 | 0 | 1 | 2 |
| 62 | ASD | 0 | 0 | 1 | 0 | 0 | 1 |
| 63 | ASD | 0 | 0 | 0 | 0 | 0 | 2 |
| 64 | ASD | 0 | 0 | 0 | 0 | 0 | 1 |
| 65 | ASD | 0 | 1 | 0 | 0 | 0 | 4 |
| 66 | ASD | 1 | 1 | 0 | 0 | 0 | 3 |
| 67 | ASD | 0 | 1 | 0 | 1 | 1 | 5 |
| 68 | ASD | 1 | 0 | 1 | 1 | 0 | 8 |
| 69 | ASD | 0 | 0 | 0 | 0 | 0 | 2 |
| 70 | ASD | 0 | 1 | 0 | 0 | 1 | 2 |
| 71 | ASD | 0 | 1 | 0 | 0 | 0 | 1 |
| 72 | ASD | 1 | 0 | 0 | 0 | 0 | 2 |
| 73 | ASD | 0 | 0 | 0 | 0 | 0 | 2 |
| 74 | ASD | 0 | 0 | 1 | 0 | 0 | 3 |
| 75 | ASD | 0 | 1 | 1 | 0 | 1 | 5 |
| 76 | ASD | 1 | 1 | 1 | 0 | 0 | 8 |
| 77 | ASD | 1 | 1 | 0 | 1 | 0 | 5 |
| 78 | ASD | 1 | 1 | 0 | 0 | 0 | 4 |
| 79 | ASD | 0 | 1 | 0 | 0 | 0 | 3 |
| 80 | ASD | 0 | 1 | 0 | 0 | 0 | 2 |
| 81 | ASD | 0 | 0 | 0 | 0 | 0 | 0 |
| 82 | ASD | 0 | 1 | 0 | 0 | 0 | 1 |
| 83 | ASD | 0 | 1 | 0 | 0 | 0 | 3 |
| 84 | ASD | 0 | 0 | 1 | 0 | 0 | 7 |
| 85 | ASD | 0 | 0 | 0 | 0 | 0 | 1 |
| 86 | ASD/GJH | 0 | 0 | 0 | 1 | 0 | 6 |
| 87 | ASD/GJH | 0 | 1 | 1 | 0 | 0 | 4 |
| 88 | ASD/GJH | 1 | 1 | 0 | 0 | 0 | 4 |
| 89 | ASD/GJH | 0 | 0 | 0 | 1 | 0 | 4 |
| 90 | ASD/GJH | 0 | 0 | 0 | 0 | 0 | 0 |
| 91 | ASD/GJH | 0 | 0 | 0 | 0 | 0 | 3 |
| 92 | ASD/GJH | 0 | 0 | 0 | 0 | 0 | 2 |
| 93 | ASD/GJH | 0 | 1 | 1 | 1 | 0 | 8 |
| 94 | ASD/GJH | 0 | 0 | 1 | 1 | 0 | 10 |
| 95 | ASD/GJH | 0 | 0 | 1 | 0 | 0 | 4 |
| 96 | ASD/GJH | 1 | 1 | 1 | 0 | 0 | 6 |
| 97 | ASD/GJH | 0 | 1 | 0 | 1 | 1 | 8 |
| 98 | ASD/GJH | 1 | 0 | 0 | 1 | 0 | 8 |
| 99 | ASD/GJH | 0 | 0 | 0 | 0 | 0 | 1 |
| 100 | ASD/GJH | 0 | 1 | 0 | 0 | 0 | 4 |
| 101 | ASD/GJH | 1 | 1 | 0 | 1 | 0 | 9 |
| 102 | ASD/GJH | 0 | 0 | 0 | 1 | 0 | 2 |
| 103 | ASD/GJH | 0 | 0 | 0 | 0 | 0 | 3 |
| 104 | ASD/GJH | 1 | 1 | 1 | 1 | 0 | 8 |
| 105 | ASD/GJH | 1 | 1 | 0 | 0 | 0 | 7 |

**Supplementary Table 6.** Sum of immune symptoms by age range (child, teen, and adult). 0 = no symptom reported; 1 = symptom reported.

| **ID#** | **Group** | **Child Immune** | **Teen Immune** | **Adult Immune** |
| --- | --- | --- | --- | --- |
| 1 | ASD | 0 | 0 | 0 |
| 2 | ASD | 0 | 1 | 0 |
| 3 | ASD | 0 | 0 | 0 |
| 4 | ASD | 0 | 1 | 0 |
| 5 | ASD | 2 | 1 | 1 |
| 6 | ASD | 1 | 0 | 1 |
| 7 | ASD | 1 | 0 | 0 |
| 8 | ASD | 3 | 2 | 1 |
| 9 | ASD | 3 | 2 | 2 |
| 10 | ASD | 0 | 1 | 1 |
| 11 | ASD | 0 | 0 | 0 |
| 12 | ASD | 1 | 1 | 1 |
| 13 | ASD | 0 | 1 | 6 |
| 14 | ASD | 1 | 0 | 1 |
| 15 | ASD | 2 | 3 | 3 |
| 16 | ASD | 4 | 5 | 6 |
| 17 | ASD | 1 | 0 | 0 |
| 18 | ASD | 1 | 2 | 0 |
| 19 | ASD | 2 | 2 | 3 |
| 20 | ASD | 3 | 3 | 3 |
| 21 | ASD | 1 | 1 | 0 |
| 22 | ASD | 2 | 3 | 3 |
| 23 | ASD | 6 | 3 | 3 |
| 24 | ASD | 0 | 0 | 0 |
| 25 | ASD | 0 | 0 | 1 |
| 26 | ASD | 4 | 2 | 1 |
| 27 | ASD | 1 | 1 | 6 |
| 28 | ASD | 4 | 4 | 4 |
| 29 | ASD | 5 | 6 | 4 |
| 30 | ASD | 0 | 2 | 2 |
| 31 | ASD | 1 | 0 | 0 |
| 32 | ASD | 2 | 2 | 0 |
| 33 | ASD | 0 | 0 | 0 |
| 34 | ASD | 1 | 0 | 0 |
| 35 | ASD | 3 | 4 | 3 |
| 36 | ASD | 1 | 0 | 0 |
| 37 | ASD | 1 | 2 | 3 |
| 38 | ASD | 2 | 4 | 2 |
| 39 | ASD | 0 | 0 | 1 |
| 40 | ASD | 1 | 0 | 3 |
| 41 | ASD | 2 | 7 | 5 |
| 42 | ASD | 1 | 0 | 2 |
| 43 | ASD | 4 | 0 | 3 |
| 44 | ASD | 0 | 0 | 0 |
| 45 | ASD | 0 | 1 | 3 |
| 46 | ASD | 0 | 0 | 0 |
| 47 | ASD | 2 | 1 | 1 |
| 48 | ASD | 0 | 0 | 0 |
| 49 | ASD | 0 | 0 | 0 |
| 50 | ASD | 1 | 1 | 0 |
| 51 | ASD | 2 | 2 | 2 |
| 52 | ASD | 0 | 1 | 1 |
| 53 | ASD | 0 | 2 | 2 |
| 54 | ASD | 0 | 0 | 3 |
| 55 | ASD | 6 | 5 | 7 |
| 56 | ASD | 5 | 5 | 7 |
| 57 | ASD | 1 | 0 | 1 |
| 58 | ASD | 0 | 1 | 0 |
| 59 | ASD | 1 | 2 | 0 |
| 60 | ASD | 5 | 5 | 5 |
| 61 | ASD | 1 | 0 | 4 |
| 62 | ASD | 5 | 5 | 7 |
| 63 | ASD | 3 | 3 | 4 |
| 64 | ASD | 3 | 4 | 4 |
| 65 | ASD | 1 | 1 | 0 |
| 66 | ASD | 0 | 1 | 2 |
| 67 | ASD | 6 | 5 | 2 |
| 68 | ASD | 5 | 4 | 4 |
| 69 | ASD | 6 | 6 | 6 |
| 70 | ASD | 0 | 0 | 0 |
| 71 | ASD | 4 | 4 | 4 |
| 72 | ASD | 2 | 2 | 1 |
| 73 | ASD | 3 | 3 | 5 |
| 74 | ASD | 3 | 2 | 2 |
| 75 | ASD | 5 | 5 | 5 |
| 76 | ASD | 1 | 1 | 5 |
| 77 | ASD | 0 | 0 | 2 |
| 78 | ASD | 1 | 0 | 0 |
| 79 | ASD | 5 | 7 | 7 |
| 80 | ASD | 2 | 5 | 7 |
| 81 | ASD | 6 | 5 | 5 |
| 82 | ASD | 1 | 0 | 0 |
| 83 | ASD | 1 | 0 | 3 |
| 84 | ASD | 0 | 0 | 1 |
| 85 | ASD | 0 | 1 | 1 |
| 86 | ASD/GJH | 2 | 3 | 2 |
| 87 | ASD/GJH | 5 | 7 | 8 |
| 88 | ASD/GJH | 4 | 3 | 3 |
| 89 | ASD/GJH | 3 | 3 | 2 |
| 90 | ASD/GJH | 4 | 3 | 3 |
| 91 | ASD/GJH | 1 | 3 | 4 |
| 92 | ASD/GJH | 7 | 4 | 6 |
| 93 | ASD/GJH | 3 | 3 | 2 |
| 94 | ASD/GJH | 5 | 6 | 6 |
| 95 | ASD/GJH | 1 | 1 | 5 |
| 96 | ASD/GJH | 0 | 0 | 2 |
| 97 | ASD/GJH | 1 | 0 | 2 |
| 98 | ASD/GJH | 3 | 2 | 6 |
| 99 | ASD/GJH | 5 | 4 | 4 |
| 100 | ASD/GJH | 3 | 3 | 5 |
| 101 | ASD/GJH | 5 | 6 | 8 |
| 102 | ASD/GJH | 1 | 1 | 0 |
| 103 | ASD/GJH | 0 | 0 | 2 |
| 104 | ASD/GJH | 2 | 2 | 4 |
| 105 | ASD/GJH | 3 | 0 | 0 |

**Supplementary Table 7.** Symptoms of irritable bowel syndrome/gastrointestinal dysmotility (IBS), joint pain, other chronic pain, epilepsy, and birth control/hormone treatment (BC Tx). 0 = no symptom reported; 1 = symptom reported.

| **ID#** | **Group** | **IBS** | **Joint Pain** | **Other Chronic Pain** | **Epilepsy** | **BC Tx** |
| --- | --- | --- | --- | --- | --- | --- |
| 1 | ASD | 0 | 0 | 2 | 0 | 0 |
| 2 | ASD | 0 | 0 | 2 | 0 | 0 |
| 3 | ASD | 1 | 0 | 2 | 0 | 0 |
| 4 | ASD | 0 | 0 | 2 | 0 | 0 |
| 5 | ASD | 1 | 0 | 2 | 0 | 0 |
| 6 | ASD | 1 | 1 | 2 | 0 | 0 |
| 7 | ASD | 1 | 1 | 2 | 0 | 0 |
| 8 | ASD | 0 | 0 | 1 | 0 | 0 |
| 9 | ASD | 1 | 1 | 2 | 0 | 0 |
| 10 | ASD | 0 | 0 | 2 | 0 | 0 |
| 11 | ASD | 0 | 1 | 2 | 0 | 0 |
| 12 | ASD | 0 | 0 | 2 | 0 | 0 |
| 13 | ASD | 0 | 1 | 2 | 1 | 0 |
| 14 | ASD | 1 | 0 | 3 | 0 | 0 |
| 15 | ASD | 1 | 0 | 2 | 0 | 0 |
| 16 | ASD | 1 | 1 | 1 | 0 | 1 |
| 17 | ASD | 1 | 0 | 2 | 0 | 0 |
| 18 | ASD | 1 | 1 | 1 | 0 | 0 |
| 19 | ASD | 1 | 1 | 1 | 0 | 0 |
| 20 | ASD | 1 | 1 | 4 | 0 | 0 |
| 21 | ASD | 0 | 0 | 2 | 0 | 0 |
| 22 | ASD | 1 | 0 | 2 | 0 | 0 |
| 23 | ASD | 1 | 0 | 2 | 0 | 0 |
| 24 | ASD | 1 | 0 | 2 | 0 | 1 |
| 25 | ASD | 1 | 0 | 2 | 0 | 1 |
| 26 | ASD | 0 | 1 | 3 | 0 | 0 |
| 27 | ASD | 1 | 1 | 1 | 0 | 1 |
| 28 | ASD | 1 | 0 | 1 | 1 | 1 |
| 29 | ASD | 0 | 0 | 2 | 0 | 0 |
| 30 | ASD | 1 | 0 | 4 | 0 | 1 |
| 31 | ASD | 1 | 0 | 4 | 1 | 1 |
| 32 | ASD | 0 | 0 | no response | 0 | 1 |
| 33 | ASD | 1 | 0 | 2 | 0 | 0 |
| 34 | ASD | 1 | 0 | 2 | 0 | 0 |
| 35 | ASD | 1 | 0 | 2 | 0 | 0 |
| 36 | ASD | 0 | 0 | 2 | 0 | 0 |
| 37 | ASD | 0 | 0 | 2 | 0 | 1 |
| 38 | ASD | 1 | 0 | 1 | 1 | 0 |
| 39 | ASD | 1 | 0 | 1 | 0 | 1 |
| 40 | ASD | 1 | 0 | 2 | 0 | 0 |
| 41 | ASD | 0 | 1 | 1 | 0 | 0 |
| 42 | ASD | 1 | 0 | 1 | 0 | 0 |
| 43 | ASD | 0 | 0 | 1 | 0 | 0 |
| 44 | ASD | 0 | 0 | 2 | 0 | 0 |
| 45 | ASD | 1 | 0 | 2 | 0 | 0 |
| 46 | ASD | 1 | 0 | 2 | 0 | 0 |
| 47 | ASD | 0 | 0 | 2 | 0 | 1 |
| 48 | ASD | 0 | 0 | 2 | 0 | 0 |
| 49 | ASD | 1 | 0 | 2 | 0 | 0 |
| 50 | ASD | 1 | 1 | 1 | 0 | 0 |
| 51 | ASD | 1 | 0 | 2 | 0 | 0 |
| 52 | ASD | 1 | 0 | 2 | 0 | 0 |
| 53 | ASD | 1 | 0 | 2 | 0 | 0 |
| 54 | ASD | 1 | 1 | 1 | 0 | 0 |
| 55 | ASD | 1 | 1 | 1 | 0 | 0 |
| 56 | ASD | 1 | 0 | 1 | 1 | 0 |
| 57 | ASD | 0 | 0 | 2 | 0 | 0 |
| 58 | ASD | 1 | 0 | 2 | 0 | 1 |
| 59 | ASD | 0 | 0 | 2 | 0 | 0 |
| 60 | ASD | 0 | 1 | 2 | 0 | 1 |
| 61 | ASD | 1 | 1 | 2 | 0 | 0 |
| 62 | ASD | 1 | 0 | 1 | 0 | 0 |
| 63 | ASD | 0 | 0 | 2 | 0 | 0 |
| 64 | ASD | 1 | 1 | 1 | 0 | 0 |
| 65 | ASD | 1 | 0 | 2 | 0 | 0 |
| 66 | ASD | 0 | 0 | 4 | 0 | 0 |
| 67 | ASD | 0 | 1 | 1 | 0 | 0 |
| 68 | ASD | 1 | 1 | 2 | 0 | 1 |
| 69 | ASD | 1 | 1 | 1 | 0 | 0 |
| 70 | ASD | 0 | 0 | 1 | 0 | 0 |
| 71 | ASD | 1 | 0 | 2 | 0 | 0 |
| 72 | ASD | 0 | 0 | 2 | 0 | 0 |
| 73 | ASD | 1 | 0 | no response | 0 | 0 |
| 74 | ASD | 1 | 1 | 2 | 0 | 0 |
| 75 | ASD | 0 | 0 | 1 | 0 | 0 |
| 76 | ASD | 1 | 1 | 1 | 0 | 0 |
| 77 | ASD | 1 | 0 | 1 | 0 | 1 |
| 78 | ASD | 0 | 0 | 2 | 0 | 0 |
| 79 | ASD | 1 | 1 | 1 | 0 | 1 |
| 80 | ASD | 1 | 0 | 1 | 0 | 0 |
| 81 | ASD | 0 | 0 | 2 | 0 | 1 |
| 82 | ASD | 1 | 0 | 3 | 0 | 0 |
| 83 | ASD | 0 | 0 | 3 | 0 | 0 |
| 84 | ASD | 1 | 1 | 1 | 0 | 1 |
| 85 | ASD | 0 | 0 | 2 | 0 | 0 |
| 86 | ASD/GJH | 1 | 1 | 1 | 0 | 1 |
| 87 | ASD/GJH | 1 | 1 | 1 | 1 | 0 |
| 88 | ASD/GJH | 1 | 1 | 1 | 1 | 0 |
| 89 | ASD/GJH | 1 | 1 | 1 | 0 | 0 |
| 90 | ASD/GJH | 0 | 1 | 2 | 0 | 0 |
| 91 | ASD/GJH | 0 | 1 | 2 | 0 | 1 |
| 92 | ASD/GJH | 1 | 1 | 1 | 0 | 1 |
| 93 | ASD/GJH | 1 | 1 | 1 | 0 | 1 |
| 94 | ASD/GJH | 1 | 1 | 1 | 0 | 0 |
| 95 | ASD/GJH | 0 | 1 | 2 | 0 | 0 |
| 96 | ASD/GJH | 1 | 1 | 1 | 0 | 0 |
| 97 | ASD/GJH | 1 | 1 | 3 | 0 | 0 |
| 98 | ASD/GJH | 1 | 1 | 1 | 0 | 1 |
| 99 | ASD/GJH | 1 | 1 | 2 | 0 | 0 |
| 100 | ASD/GJH | 1 | 1 | 1 | 0 | 1 |
| 101 | ASD/GJH | 1 | 1 | 1 | 0 | 1 |
| 102 | ASD/GJH | 0 | 1 | 1 | 0 | 1 |
| 103 | ASD/GJH | 0 | 1 | 1 | 0 | 0 |
| 104 | ASD/GJH | 1 | 1 | 1 | 0 | 1 |
| 105 | ASD/GJH | 1 | 1 | 1 | 0 | 0 |
